# Group 1 CD1-restricted T cells contribute to control of systemic *Staphylococcus aureus* infection

**DOI:** 10.1371/journal.ppat.1008443

**Published:** 2020-04-28

**Authors:** Lavanya Visvabharathy, Samantha Genardi, Liang Cao, Ying He, Francis Alonzo, Evgeny Berdyshev, Chyung-Ru Wang

**Affiliations:** 1 Department of Microbiology and Immunology, Feinberg School of Medicine, Northwestern University, Chicago, United States of America; 2 Department of Microbiology and Immunology, Stritch School of Medicine, Loyola University, Maywood, United States of America; 3 Department of Medicine, National Jewish Health, Denver, United States of America; Portland VA Medical Center, Oregon Health and Science University, UNITED STATES

## Abstract

*Staphylococcus aureus* (SA) is the causative agent of both skin/soft tissue infections as well as invasive bloodstream infections. Though vaccines have been developed to target both humoral and T cell-mediated immune responses against SA, they have largely failed due to lack of protective efficacy. Group 1 CD1-restricted T cells recognize lipid rather than peptide antigens. Previously found to recognize lipids derived from cell wall of *Mycobacterium tuberculosis* (Mtb), these cells were associated with protection against Mtb infection in humans. Using a transgenic mouse model expressing human group 1 CD1 molecules (hCD1Tg), we demonstrate that group 1 CD1-restricted T cells can recognize SA-derived lipids in both immunization and infection settings. Systemic infection of hCD1Tg mice showed that SA-specific group 1 CD1-restricted T cell response peaked at 10 days post-infection, and hCD1Tg mice displayed significantly decreased kidney pathology at this time point compared with WT control mice. Immunodominant SA lipid antigens recognized by group 1 CD1-restricted T cells were comprised mainly of cardiolipin and phosphatidyl glycerol, with little contribution from lysyl-phosphatidyl glycerol which is a unique bacterial lipid not present in mammals. Group 1 CD1-restricted T cell lines specific for SA lipids also conferred protection against SA infection in the kidney after adoptive transfer. They were further able to effectively control SA replication in vitro through direct antigen presentation by group 1 CD1-expressing BMDCs. Together, our data demonstrate a previously unknown role for group 1 CD1-restricted SA lipid-specific T cells in the control of systemic MRSA infection.

## Author summary

The bacterial pathogen *Staphylococcus aureus* (SA) is the cause of skin as well as invasive bloodstream infections, both in hospital and community settings. Efforts to develop preventative vaccines have been unsuccessful so far despite focusing on stimulating both B cell and T cell responses against SA. However, the unconventional group 1 CD1-restricted lipid-reactive T cell subset has never been studied in the context of systemic SA infection, and investigating their contribution to SA host defense could be fruitful in anti-SA vaccine design. Using a mouse model expressing group 1 CD1 molecules, we show that these T cells recognize and are activated by SA lipids during immunization and infection. The presence of group 1 CD1-restricted T cells decrease kidney inflammation in mice, and transfer of these cells into mice before SA infection results in ~10,000-fold decreases in kidney bacterial burdens. Together, our data demonstrate that activation of the group 1 CD1-restricted T cell response may be a promising avenue to investigate in the development of anti-SA vaccines.

## Introduction

*Staphylococcus aureus* (SA) is one of the most frequently isolated pathogens associated with nosocomial infections [1]. SA infection commonly manifests in many different organ niches, including skin/soft tissue infections, respiratory tract infections, pneumonia, and endocarditis [2]. SA also causes invasive sepsis leading to septic shock and multiple organ failure [3–6]. Antibiotic treatment of SA is complicated by the prevalence of methicillin resistant SA (MRSA) strains in hospitals and increasingly in community settings [3] [7], resulting in patient mortality rates of 16.6% in 2017 in addition to elevated healthcare costs [8–10]. Multiple vaccine strategies have been employed with limited efficacy against recurrent infection [11–13].

As an extracellular pathogen, historically it was thought that the antibody response was critical to immunity against SA [14, 15]. However, the contribution of T cells to anti-SA immunity is gradually becoming appreciated. One study identified a CD4^+^ T cell epitope from the SA heme-uptake protein IsdB able to induce proliferation and IFN-γ and IL-17 secretion from T cells [16]. Another group identified the epitope M8 derived from manganese transport protein C (MntC) to induce proliferation and IL-17 secretion from CD4^+^ T cells in immunized BALB/c mice [17]. Studies in humans without documented prior infection with SA showed that healthy people possess a significant pool of SA Ag-specific CD4^+^ memory T cells [18], and mouse studies have linked expansion of IL-17-producing γδ T cells with protection against SA [19]. Additionally, patients with hyper-IgE syndrome who lack the ability for Th17 CD4^+^ T cell differentiation are highly susceptible to SA skin infections [20], and HIV^+^ patients are more susceptible to MRSA skin infections possibly due to decreased IFN-γ production from SA Ag-specific CD4^+^ T cells [21]. Intriguingly, recent studies have found a clonal Vγ6^+^Vδ4^+^ T cell subset secreting IL-17 to be crucial for protective immunity against SA skin infection in mice [22]. Despite data showing the importance of conventional CD4^+^ T cells and γδ T cells to defense against SA, the role of lipid antigen-specific group 1 CD1-restricted T cells remains unexplored.

CD1 molecules are structurally similar to MHC-I molecules and present self- and microbial-derived lipids to T cells [23]. Three major groups of CD1 have been identified in humans: group 1 CD1 (CD1a, -b, and -c), group 2 CD1 (CD1d), and group 3 CD1 (CD1e; aids in loading complex lipids onto CD1b molecules) [24, 25]. CD1d is the restriction element for type I and type II NKT cells, innate-like T cells which have been shown to play a role in defense against various microbial infections [26–28]. NKT cells exhibit pre-activated phenotype and are rapidly mobilized to fight microbial infections [29]. Studies have demonstrated that type I NKT cells (or iNKT cells) can be activated by both antigen-specific and cytokine-driven mechanisms, for example through interaction of *Salmonella Typhimurium*-derived LPS with TLRs on antigen presenting cells, inducing production of IL-12 [30]. Additionally, activation of type II NKT cells has been shown to protect against SA-mediated sepsis in mouse models of infection [31]. However, though both group 1 and group 2 CD1-restricted T cells are activated by lipid antigens, it is unknown whether the kinetics and function of group 1 CD1-restricted T cell responses to SA mimic the innate-like responses seen with NKT cells. Though mice only express CD1d, our lab has developed a transgenic mouse model (hCD1Tg) expressing human group 1 and group 3 CD1 molecules in a tissue specific, physiologically relevant manner [32]. To date, studies have shown that group 1 CD1-restricted T cells can recognize and respond to mycobacterial lipid antigens during infection in hCD1Tg mice [32, 33]. In addition, Mtb lipid-specific group 1 CD1-restricted T cells contribute to anti-Mtb immunity through increased IFN-γ cytokine secretion [23, 33, 34], and Mtb patients have higher numbers of these T cells in circulation [35]. While Group 1 CD1-restricted microbial antigens identified thus far have mainly been of mycobacterial origin [25, 34, 36], CD1 molecules may bind lipid antigens from a broad range of pathogens because their antigen binding grooves can accommodate a variety of lipid species [37, 38]. Indeed, humans have CD1b-restricted T cells that recognize lipids from *Staphylococcus aureus*, *Brucella melitensis*, *Salmonella Typhimurium*, and *Borrelia burgdorferi* [39, 40].

To determine how group 1 CD1-restricted T cell dynamics and function change in response to different infectious agents, we selected SA as a model pathogen. SA contains microbial lipid moieties [41] sharing several common features with other group 1 CD1-binding antigens. Indeed, one recent study showed that CD1b dextramers loaded with phosphatidylglycerol (PG) from SA can identify and cross-react with autoreactive group 1 CD1-restricted T cells in humans [39]. Based on these studies, it is important to identify whether group 1 CD1-restricted T cells play a similar role in anti-SA immunity in vivo.

Using in vivo models of systemic SA infection in hCD1Tg mice, we found that group 1 CD1-restricted T cell responses against SA lipids can be detected in both immunization and infection settings. The kinetics of group 1 CD1-restricted T cell activation more closely resembled conventional T cell rather than NKT cell activation patterns, and hCD1Tg mice trended towards decreased kidney CFU burden and pathology compared with Tg- WT littermate controls. Fractionation of SA lipids revealed cardiolipins and polar phospholipids to be immunodominant lipid moeities recognized by group 1 CD1-restricted T cells. Furthermore, T cell lines specific for these lipids were protective against SA infection in hCD1Tg mice. SA killing experiments with group 1 CD1-restricted T cell lines specific for SA lipids also resulted in efficient CFU control in vitro. These findings demonstrate that group 1 CD1-restricted T cells can respond to SA lipids and that they contribute to protection against SA infection.

## Results

### Immunization of hCD1Tg mice with SA lipids activates group 1 CD1-restricted T cells

**Fig 1:**
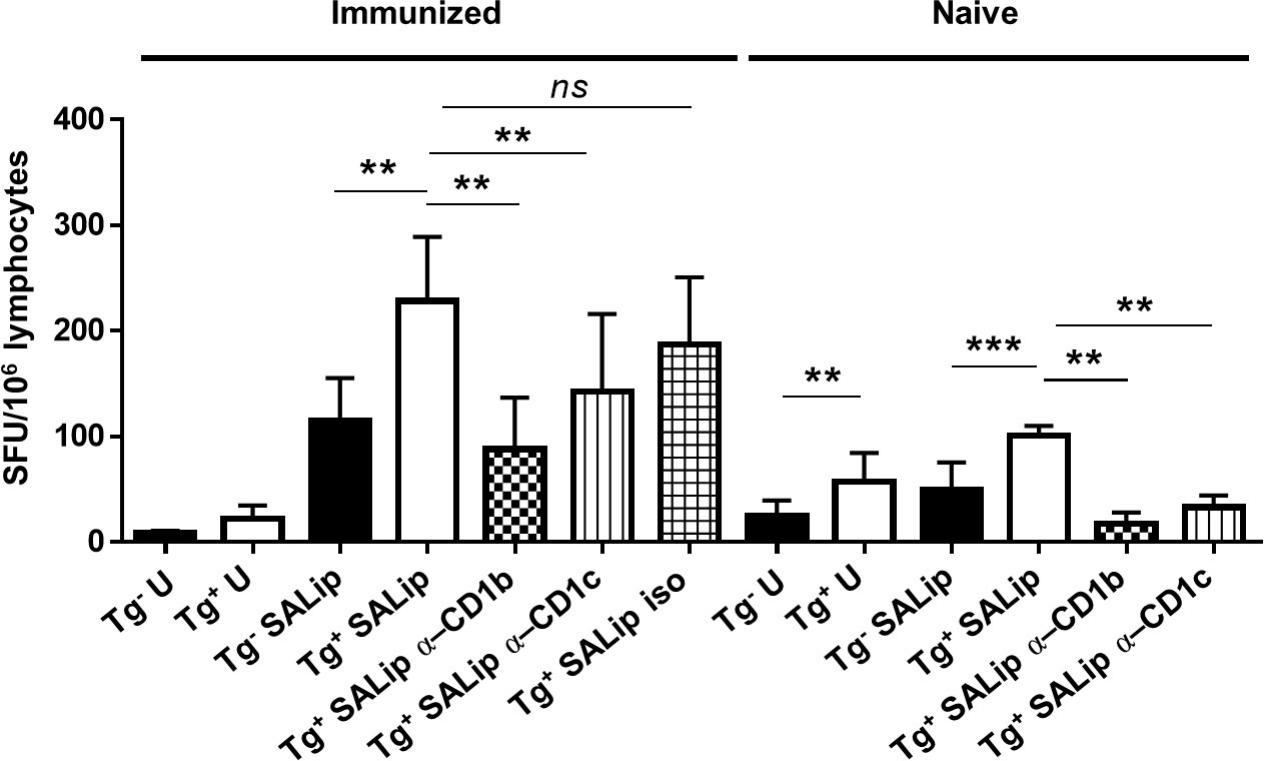
Group 1 CD1-restricted T cell responses against SA lipids are detected after immunization of hCD1Tg mice. hCD1Tg mice expressing CD1b and CD1c were immunized i.p. with Tg^+^ BMDCs coated with total SA lipid extract. Lymphocytes from pooled peripheral lymph nodes (cervical, axillary, inguinal, and mesenteric) were isolated at day 7 from immunized and naïve hCD1Tg mice. Lymphocytes were assayed for IFN-γ production via ELISPOT using Tg^-^ or Tg^+^ BMDCs ± total SA lipid as stimulators. Anti-CD1b or -CD1c blocking antibody or isotype control was added as indicated. Data is representative of 2 independent experiments with n = 1 (naïve) or 5 (immunized) mice each plated in duplicate. **p<0.01 using two-way ANOVA with Tukey’s multiple comparisons posttest.

We have previously shown that both infection with Mtb and immunization with Mtb lipids elicit group 1 CD1-restricted T cell responses in hCD1Tg mice. However, it was unknown whether group 1 CD1-restricted T cells could respond to SA lipids in hCD1Tg mice. As a proof of principle, we immunized hCD1Tg mice intraperitoneally with BMDCs expressing CD1b and CD1c (78^+^Tg, or Tg^+^ BMDCs) coated with total SA lipid extract from USA300 LAC. At 7 days post immunization, lymphocytes from peripheral lymph nodes of immunized mice were stimulated ex vivo with Tg^-^ or Tg^+^ BMDC pulsed or unpulsed with SA lipids, and IFN-γ-producing cells were quantified by ELISPOT assays. A significantly higher number of IFN-γ-producing cells were detected when stimulated with SA lipid-pulsed Tg^+^ DC compared to stimulation with SA lipid-pulsed Tg^-^ DCs or unpulsed DCs. This data indicate that SA lipid immunization can induce group 1 CD1-restricted SA lipid-specific T cell responses in hCD1Tg mice (Fig 1). Further, addition of CD1b and CD1c blocking antibodies showed that the majority of the group 1 CD1 response to SA lipids was CD1b-restricted, with a moderate contribution from CD1c-resricted T cells. Naïve, unimmunized mice also displayed a group 1 CD1-restricted T cell response to SA lipids which was also predominantly CD1b-restricted, though the magnitude of the response was much lower than what was seen in immunized mice. This suggested that a portion of the CD1b-restricted T cell response to SA lipid was due to activation of cross-reactive CD1b-autoreactive T cells.

### Group 1 CD1-restricted T cell responses directed against SA lipids can be detected during systemic SA infection in hCD1Tg mice

**Fig 2:**
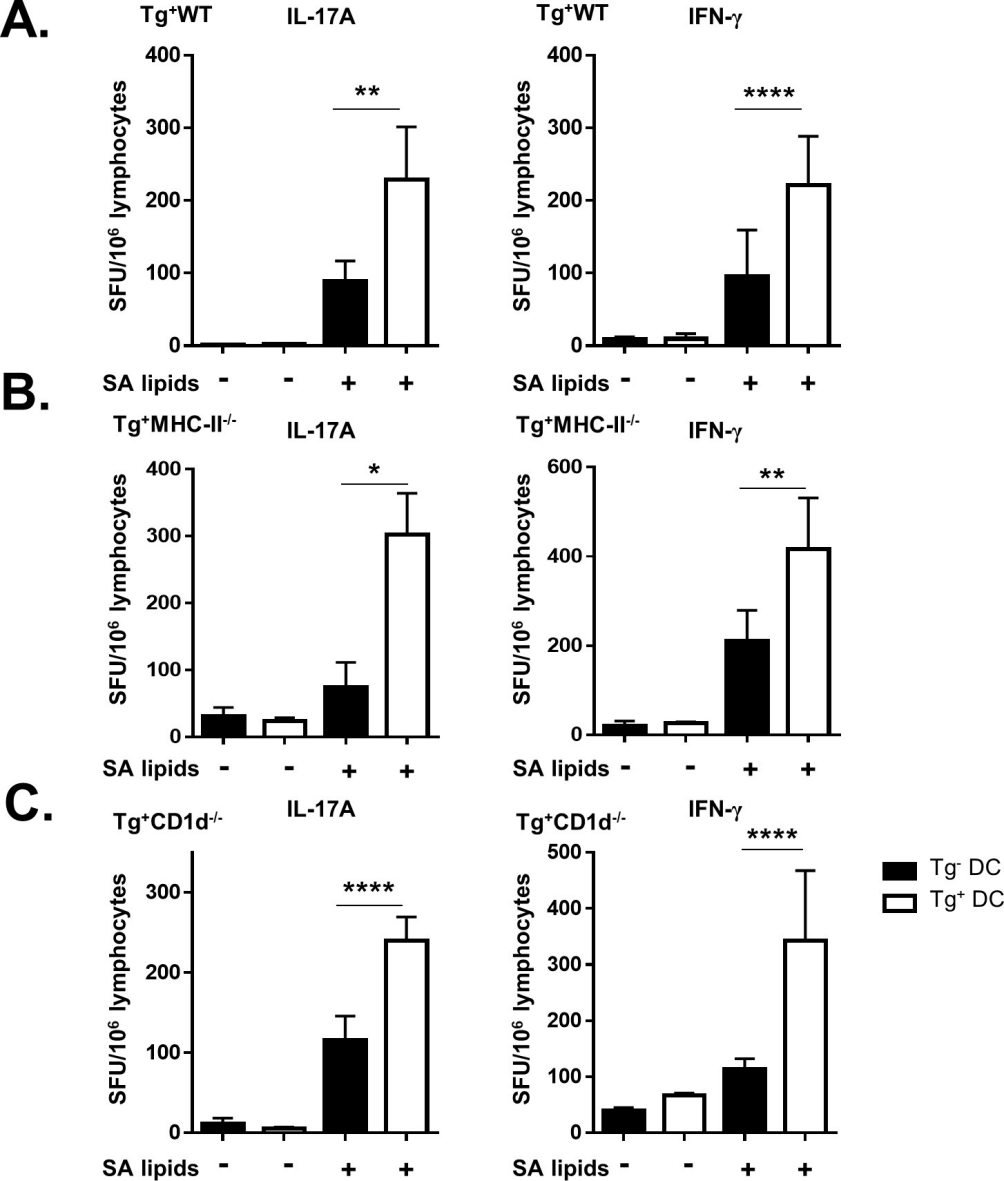
SA lipid-specific group 1 CD1-restricted T cell responses occur during systemic infection with MRSA. hCD1Tg (A) or hCD1Tg MHC-II^-/-^ (B) or hCD1Tg CD1d^-/-^ (C) mice were infected with 5x10^6^ CFU of USA300 MRSA via tail vein. At 10 days post-infection, lymphocytes from lymph nodes of indicated mice were stimulated with Tg^-^ or Tg^+^ BMDCs ± total SA lipid and IL-17A and IFN-γ-secreting cells were quantified by ELISPOT. Data representative of 3–5 independent experiments with n = 4–5 mice per group. *p<0.05, **p<0.01, ****p<0.0001 using two-way ANOVA with Tukey’s multiple comparisons posttest.

Based on data showing that immunization with SA lipids induces group 1 CD1-restricted T cell responses, we infected hCD1Tg mice with SA to determine if infection induced similar SA lipid-specific responses. hCD1Tg mice expressing CD1b and CD1c molecules (78^+^Tg mice) were infected with MRSA strain USA300 LAC, an isolate that frequently causes community-acquired skin and soft-tissue infections as well as systemic infections [42]. Mice were injected with 5x10^6^ CFU via tail vein and euthanized at 10 days post-infection. Lymphocytes were isolated from cervical, axillary, inguinal, renal, and mesenteric lymph nodes and cultured together with Tg^-^ or Tg^+^ BMDCs pulsed or unpulsed with SA lipids. This experiment was also conducted in hCD1Tg mice expressing CD1a, -b, and -c (64^+^Tg mice) and we obtained a similar magnitude of response, though there was some contribution of autoreactive CD1a-restricted T cells that was not observed in 78^+^Tg mice (S1 Fig). However, 78^+^Tg mice were used in subsequent experiments due to their ability to breed more effectively. Group 1 CD1-restricted T cells produced IL-17 and IFN-γ in response to total SA lipids in ELISPOT assays (Fig 2A). In order to confirm these responses to be SA lipid-specific and independent of non-specific superantigen-mediated T cell activation, the experiment was repeated in hCD1Tg MHC-II^-/-^ mice (Fig 2B), with similar results. We also demonstrated that the group 1 CD1-restricted T cell response to SA lipids were not affected by the presence of NKT cells, as hCD1Tg CD1d^-/-^ mice had similar levels of IFN-γ and IL-17A production as hCD1Tg WT mice (Fig 2C). In contrast to the lymph nodes, lymphocytes isolated from the spleen did not show measurable group 1 CD1-restricted SA lipid-specific responses, possibly due to the expansion of myeloid-derived suppressor cells that can limit T cell activation [43; data not shown]. We additionally determined whether SA lipid-specific group 1 CD1-restricted T cell responses were present in the kidney because it is the primary reservoir for SA during systemic infection [44]. We were unable to reliably detect these responses in the kidney at 10 days post-infection (S2 Fig), and therefore the remainder of the data focuses on group 1 CD1-restricted T cell responses in the lymph nodes. Together, these data show that group 1 CD1-restricted T cells can recognize and respond to SA lipids during systemic infection with SA.

### Group 1 CD1-restricted T cell response to SA peaks at 10 days post-infection

**Fig 3:**
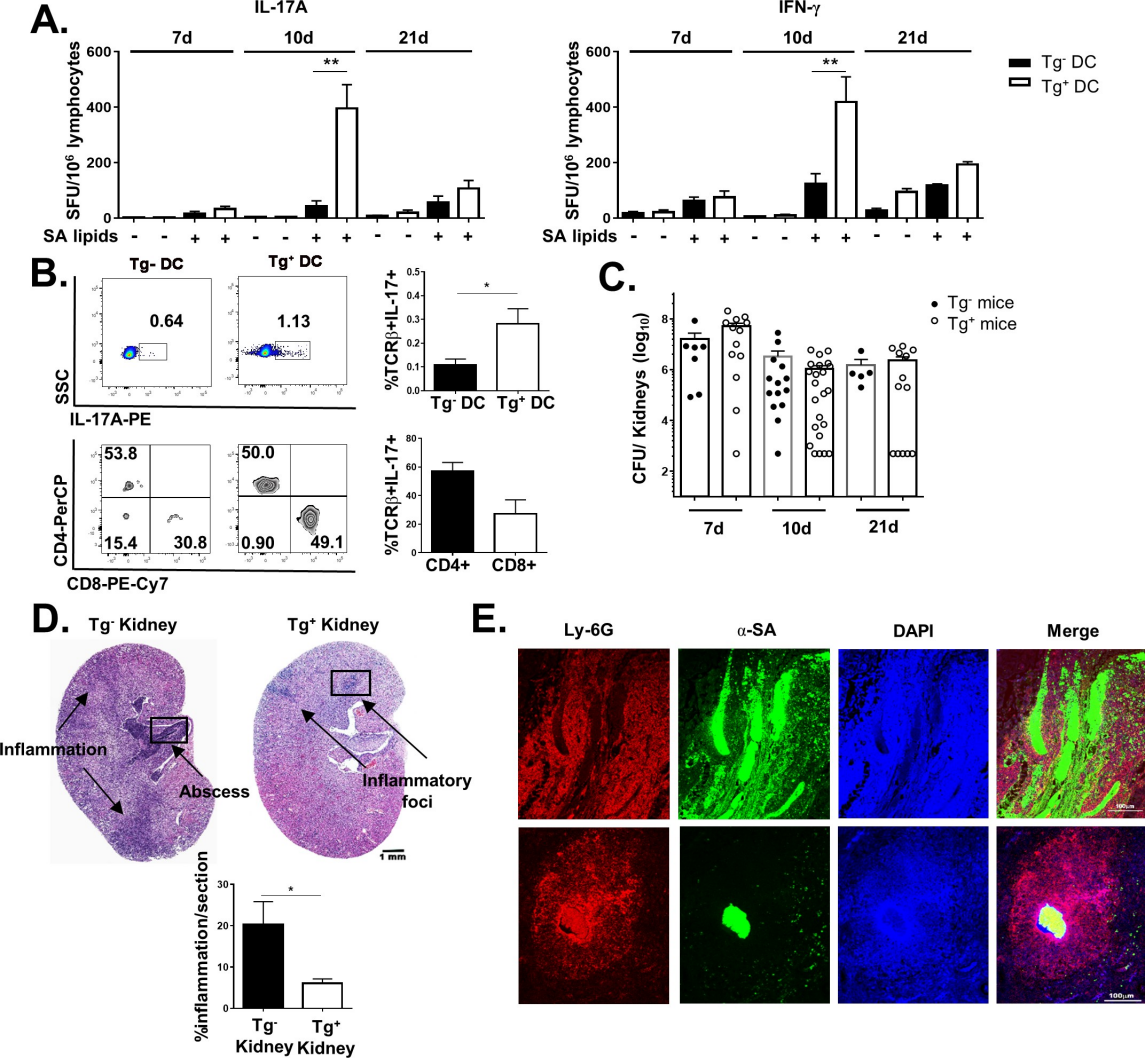
SA lipid-specific group 1 CD1-restricted T cell response helps to contain kidney inflammation. **(**A) Lymphocytes from lymph nodes of hCD1Tg mice were isolated at 7, 10, and 21 days post-infection. Frequency of IL-17A and IFN-γ-secreting cells were quantified by ELISPOT using Tg^-^ and Tg^+^ BMDC ± total SA lipids as stimulators. Data are combined from 4 independent experiments with n = 4–6 mice per time point. ** p<0.01 using 1-way ANOVA with Tukey’s posttest. (B) Enriched αβ T cells from SA-infected hCD1Tg mice at day 10 post-infection were stimulated with SA lipid pulsed Tg^-^ or Tg^+^ BMDCs. Intracellular cytokine staining for IL-17A was then performed along with surface FACS staining to determine the expression of CD4 and CD8 on SA lipid-specific group 1 CD1 T cells. Data representative of 3 independent experiments with n = 5–6 mice per experiment. *p<0.05 using two-tailed Student’s t test. (C) CFU enumeration of SA in kidneys of hCD1Tg and Tg^-^ mice at various times post-infection. Data pooled from 5 independent experiments. (D) Hematoxylin and eosin staining of hCD1Tg^+^ and Tg^-^ kidney sections at 10 days post-infection. Black boxes denote area shown in Figure E. Bar graphs quantify percentage of kidney tissue associated with inflammation. Data representative of 2 independent experiments with n = 3–6 mice per group. *p<0.05 using two-tailed Student’s t test. (E) Immunofluorescent staining was performed on paraffin-embedded kidney sections isolated from hCD1Tg mice at 10 days post-infection. Neutrophils: anti-Ly-6G, red; SA: anti-SA, green. Data representative of 2 independent experiments with n = 3–5 mice/group.

To determine the kinetics of the group 1 CD1-restricted T cell response during SA infection, hCD1Tg mice were infected i.v. with SA and sacrificed at 7, 10, and 21 days post-infection. The group 1 CD1-restricted T cell response to SA lipids peaked at 10 days, and had waned by 21 days post-infection (Fig 3A). This mirrored the activation pattern of conventional T cells, as reflected by the upregulation of CD69 expression on CD4^+^ and CD8^+^ T cells (S3A Fig). However, the peak of the group 1 CD1 response was not correlated with significant upregulation of CD1b or CD1c on APCs (e.g. B cells, macrophages, and dendritic cells) in lymph nodes of infected mice at 10dpi (S3B and S3C Fig). As the phenotypic characteristics of group 1 CD1-restricted T cells induced during SA infection are unknown, we further determined their co-receptor usage in SA-infected hCD1Tg mice. Lymphocytes from lymph nodes of infected mice were stimulated with Tg^-^ or Tg^+^ BMDCs pulsed with SA lipid antigen and intracellular cytokine staining for IL-17A was performed to identify SA lipid-specific group 1 CD1-restricted T cells (Fig 3B). We found that most of group 1 CD1-restricted SA lipid-specific T cells express either CD4 or CD8 co-receptors (Fig 3B, bottom right).

We next investigated whether the presence of group 1 CD1-restricted T cells conferred protection against systemic SA infection. Interestingly, there was a trend toward decreased CFU in kidneys of hCD1Tg mice compared with Tg^-^ littermate controls at 10 days post-infection (Fig 3C), which correlated with the peak of the group 1 CD1-restricted response to SA lipids. In addition, hCD1Tg mice exhibited significantly decreased kidney pathology compared with Tg^-^ littermate controls. Hematoxylin and eosin staining performed on infected hCD1Tg and Tg^-^ kidneys at 10 days post-infection showed that Tg^-^ kidneys displayed significantly larger areas of abscesses and inflammation (Fig 3D). Immunofluorescence on whole kidney sections further showed that abscesses were more disorganized in Tg^-^ (WT) mice compared with hCD1Tg mice at the same time point (Fig 3E). This trend toward enhanced disorganization in neutrophil recruitment and abscess formation occurred across most of the Tg^-^ kidneys examined (S4 Fig). In contrast, Tg^+^ kidneys had small, defined abscesses and foci of inflammation which limited the total area of kidney damage.

### Fractionation of SA lipids reveals immunodominant phospholipid components

**Fig 4:**
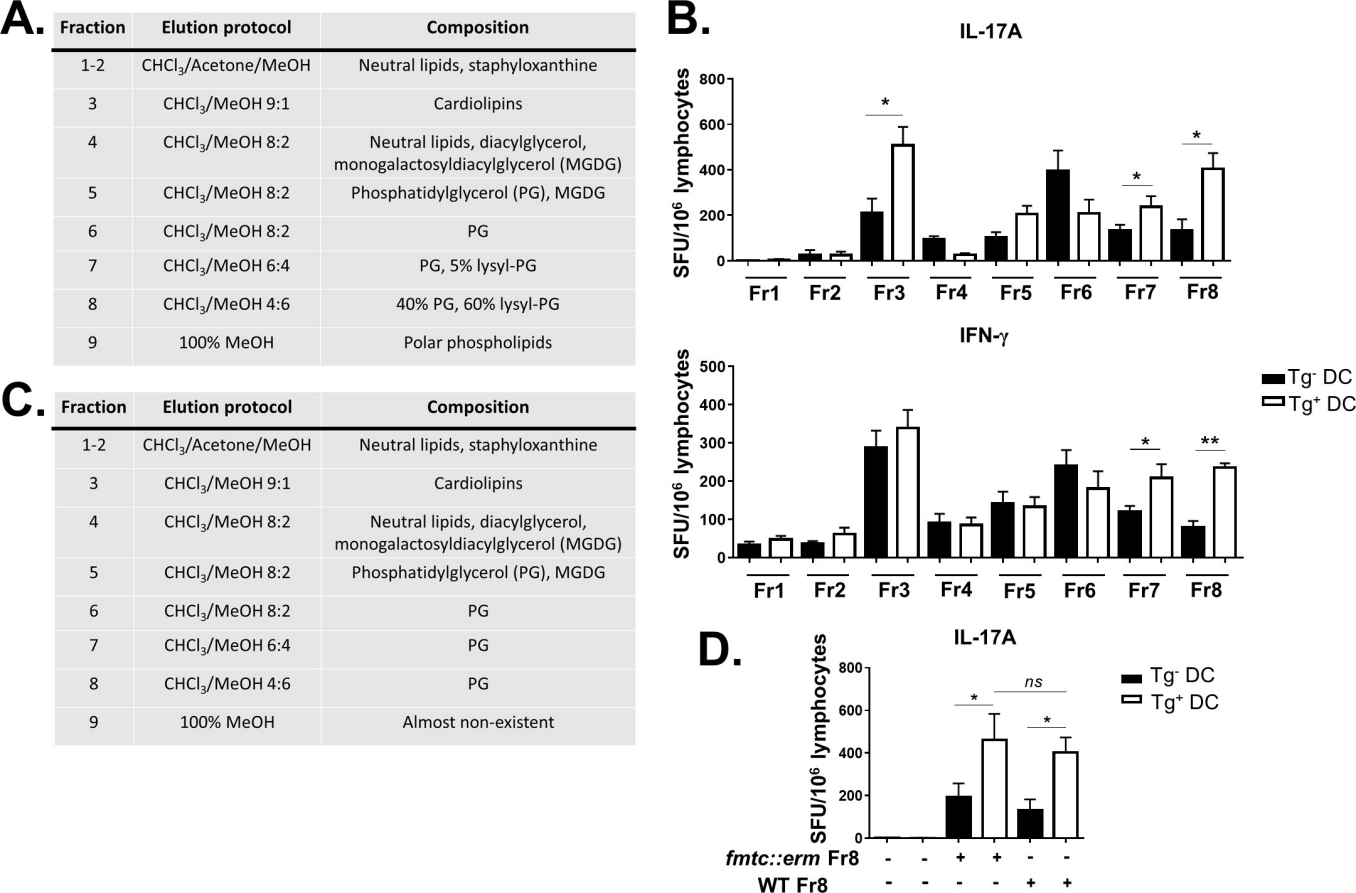
Phosphatidyl glycerol moieties are immunodominant SA lipids recognized by group 1 CD1-restricted T cells. **(**A) Lipid composition of various fractions isolated from SA using CHCl_3_-MeOH step gradients. (B) Lymphocytes from pooled peripheral lymph nodes were isolated from SA-infected hCD1Tg mice at 10 days post-infection. Frequency of IL-17A and IFN-g-producing T cells to various SA lipid fractions were quantitated by ELISPOT. Data representative of 2 independent experiments with n = 5–7 mice per experiment. (C) Table showing lipid composition of various fractions isolated from *fmtc*::*erm* USA300 strain lacking lysyl-PG. (D) Lymphocytes from hCD1Tg mice were stimulated with Tg^-^ or Tg^+^ BMDCs pulsed with Fr 8 from WT or the *fmtC*::*erm* mutant and assayed for IL-17A production as above. *p<0.05; **p<0.01 using 2-way ANOVA with Tukey’s posttest.

Having shown that SA infection can elicit SA lipid-specific group 1 CD1-restricted T cell responses, we next sought to identify immunodominant SA lipid antigens recognized during SA infection. Total SA lipids were isolated from lysostaphin-treated USA300 LAC in order to eliminate peptidoglycan contamination and limit purification to membrane-associated bacterial lipids. Lipids were fractionated using silica gel column chromatography and sequential elution of neutral lipids by chloroform, glycolipids by acetone, column re-equilibration in chloroform and by increasing methanol proportion in chloroform to elute more polar lipids. Lipids were separated into 8 discrete fractions (Fig 4A) containing neutral lipids, moderately polar lipids, and polar phospholipids. Fraction 3 contained cardiolipin species with saturated acyl chains of various chain lengths (S5A Fig). PG-enriched fractions 6–8 consisted of PG species with acyl chains lengths identical to cardiolipin in Fraction 3 (S5A Fig), but PG and lysyl-PG were present in different proportions in each fraction, causing the fractions to elute in different solvent compositions (S5B Fig). However, Fraction 8 did contain a PG moiety of unique acyl chain length (21:0/15:0; S5A Fig). IFN-γ and IL-17 ELISPOT assays readily detected group 1 CD1-restricted T cell responses directed toward fractions 7 & 8 (Fr7, Fr8; phosphatidyl glycerol-enriched) at 10 days post-infection (Fig 4B). Interestingly, group 1 CD1-restricted T cells also recognized lipids from fraction 3 (Fr3; cardiolipin-enriched), but this fraction selectively induced IL-17 and not IFN-γ production in a group 1 CD1-dependent manner.

Mass spectrometry analysis showed that Fr8 contained a mixture of phosphatidyl glycerol (PG) and lysyl-phosphatidyl glycerol (lysyl-PG) species. We originally hypothesized that lysyl-PG may play a dominant role in activating group 1 CD1-restricted T cells because lysyl-PG is unique to bacteria [45] while PG species are found in bacterial membrane as well as mitochondrial membranes in mammalian cells [46]. To test this, we generated a *fmtC*::*erm* SA mutant that is capable of synthesizing PG but is unable to lysylate it to form lysyl-PG [45]. The absence of lysyl-PG species in total lipid extract from *fmtC*::*erm* was confirmed by mass spectrometry (S6 Fig). Lipids from the *fmtC*::*erm* mutant were fractionated (Fig 4C) and tested in a IL-17 ELISPOT assay using lymphocytes from infected hCD1Tg mice at 10 days post-infection. Contrary to our hypothesis, group 1 CD1-restricted T cells retained their reactivity to Fr8 from the *fmtC*::*erm* strain (Fig 4D), suggesting that PG but not lysyl-PG species are the immunodominant lipids they recognize. Interestingly, no group 1 CD1-restricted T cell responses were detected towards purified mammalian 16:0–18:1 cardiolipin or 18:0 PG (S7 Fig), suggesting that SA-derived cardiolipin and PG species uniquely activate these T cells during systemic SA infection.

### Group 1 CD1-restricted T cell lines specific for SA lipids are polyfunctional and recognize multiple lipid species

**Fig 5:**
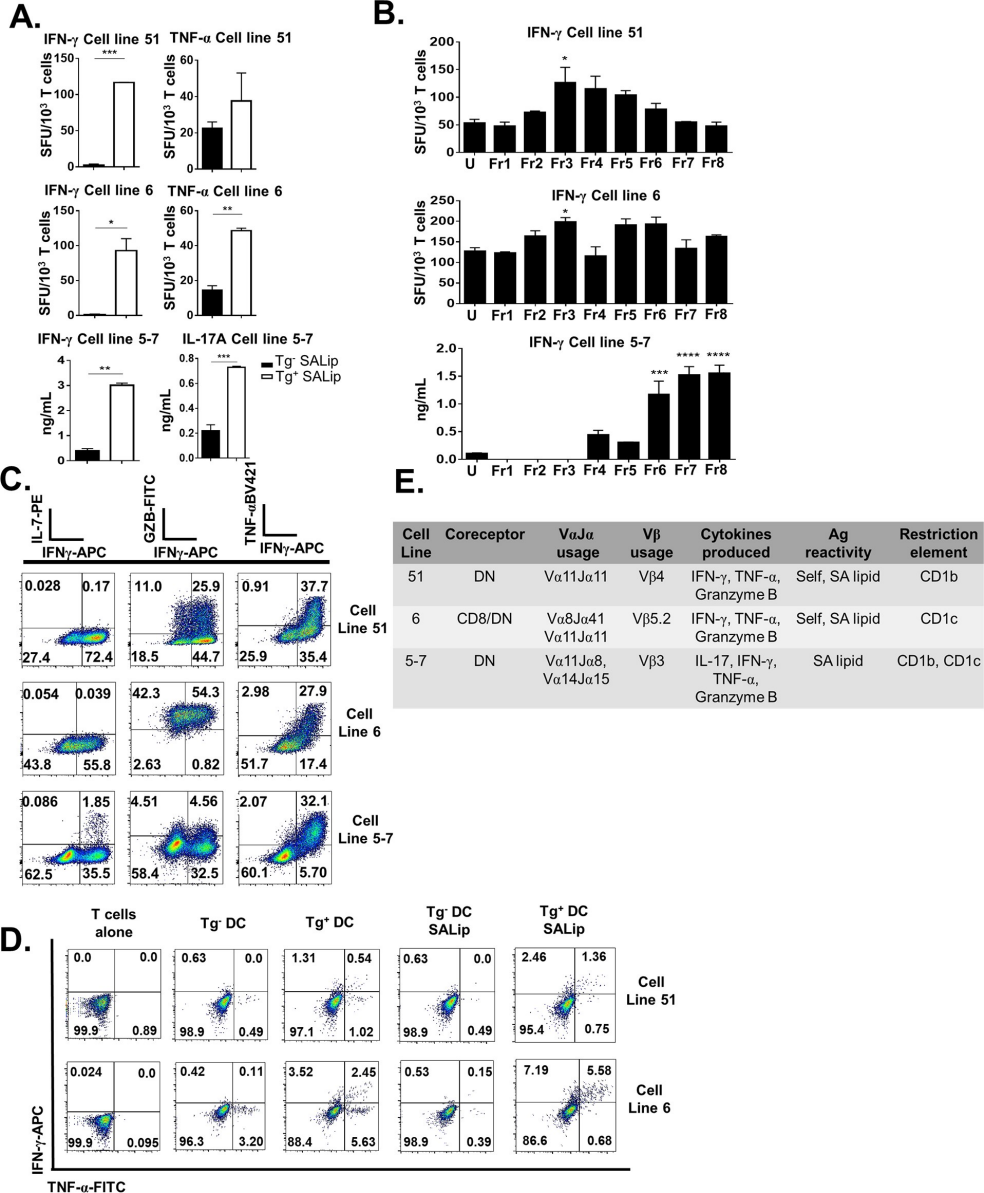
Group 1 CD1-restricted T cell lines respond to polar lipids and phosphatidyl glycerols from SA. T cell lines 51, 6 and 5–7 were derived from splenocytes of SA-infected hCD1Tg mice. (A) T cell lines were cultured with SA lipid-pulsed Tg^-^ or Tg^+^ BMDCs and assayed for IFN-γ, TNF-α, or IL-17 production by ELISA or ELISPOT as indicated. (B) T cell lines 51, 6, and 5–7 mainly recognize polar lipids and phospholipids from SA. T cells were cultured together with Tg^-^ or Tg^+^ BMDCs pulsed with the indicated SA lipid Ags for 48h (CTL 5–7) or for 18h (CTL6 & 51). Supernatants from the 5–7 T cell culture were assayed for IFN-γ production by ELISA, while IFN-γ and TNF-α ELISPOT was performed for T cell lines 6 and 51. (C) Cytokine producing capacity of 3 group 1 CD1-restricted T cell lines in response to PMA-Ionomycin stimulation. (D) Cytokine production from group 1 CD1-restricted T cell lines in response to SA lipid stimulation. (E) Table showing characteristics of 3 group 1 CD1-restricted SA lipid-specific T cell lines. Data representative of 2–3 independent experiments with n = 2 wells/condition. *p<0.05, **p<0.01, ***p<0.005 using 2-way ANOVA with Tukey’s posttest.

In order to further probe the function of SA lipid-specific group 1 CD1-restricted T cells, we generated group 1 CD1-restricted SA lipid-specific T cell lines from lymphocytes of infected hCD1Tg mice by re-stimulating them weekly with Tg^+^ BMDCs pulsed with total SA lipids. A panel of group 1 CD1-restricted T cell lines were generated and we chose to focus on 3 cell lines in particular due to their specificity and ability to produce multiple cytokines. All 3 T cell lines were reactive to SA lipids and could produce IFN-γ as well as either IL-17 or TNF-α (Fig 5A). These responses were truly SA lipid-specific and Tg-dependent, as SA lipid pulsing did not affect BMDC maturation or IL-6 production differentially between Tg^-^ and Tg^+^ BMDCs alone (S8 Fig). They also showed different immunodominance hierarchies in response to SA lipids (Fig 5B). All 3 cell lines were poly-functional and could produce TNF-α, IFN-γ, and granzyme B in response to PMA and Ionomycin stimulation; cell line 5–7 was unique in that it also produced IL-17 (Fig 5C). These group 1 CD1-restricted T cell lines were also poly-functional in response to SA lipids (Fig 5D). Each T cell line was double negative (possibly due to loss of co-receptor after long term primary T cell culture) used different Vβ chains, and most were CD1b-restricted (Fig 5E). Though all 3 cell lines used the Vα11 chain, sequence analysis demonstrated that J region rearrangements were different among them (Fig 5E). Finally, cytokine production from each cell line was likely dependent upon TCR activation by group 1 CD1 molecules, as IFN-γ production was retained when T cells were simulated by APCs lacking the critical innate immune signaling adaptor protein MyD88 (S9 Fig).

### Adoptive transfer of CD1b-restricted T cells specific for SA lipids protects against SA infection in hCD1Tg mice

**Fig 6:**
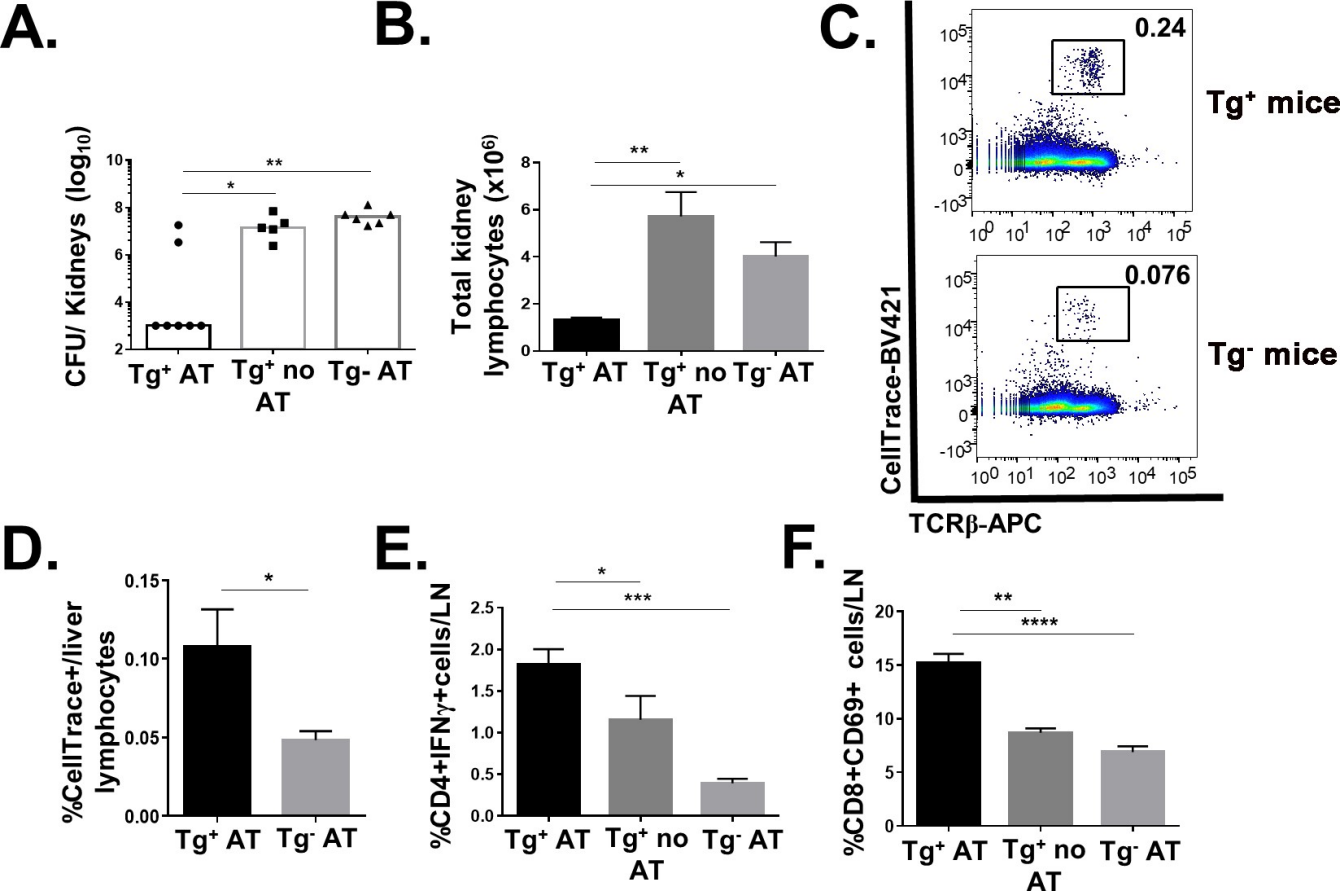
Adoptive transfer of SA lipid-specific CD1b-restricted T cells protect against SA infection in hCD1Tg mice. **(**A) PBS or 2x10^6^ cells of T cell line 51 labeled with CellTrace were adoptively transferred via tail vein into hCD1Tg or Tg^-^ mice 24h prior to infection. Mice were then infected with 2.5x10^6^ CFU of SA also via tail vein and euthanized at 4 days post-infection. Kidneys were isolated for CFU enumeration. (B) Total number of kidney lymphocytes isolated from each of the 3 treatment groups. (C) Representative dot plot showing T cell line 51 is present in higher numbers as TCRβ^+^CellTrace^+^ cells in livers of infected Tg^+^ mice compared to Tg^-^ mice. (D) Bar graph depicts percentage of adoptively transferred T cell line 51 (TCRβ^+^CellTrace^+^) in the liver of Tg^+^ and Tg^-^ recipient mice. (E) Peripheral lymph node lymphocytes stimulated with PMA-Ionomycin for 6 hours. Intracellular cytokine staining detected IFN-γ production from CD4^+^ lymphocytes. (F) CD69 expression on CD8^+^ T cells in peripheral lymph nodes of infected mice. Data representative of 2 independent experiments with n = 3–5 mice per group. *p<0.05; **p<0.01; ***p<0.001; ****p<0.0001.

Adoptive transfer of mycolic acid-specific CD1b-restricted T cells have been shown to confer protection against Mtb infection in hCD1Tg mice [33]. To test whether SA lipid-specific CD1b-restricted T cells were protective against SA infection, hCD1Tg mice and sex-matched Tg^-^ littermate controls were injected with either PBS or 2x10^6^ cells of T cell line 51 via tail vein. Mice were infected with SA i.v. 24h later and euthanized at 4 days post-infection. hCD1Tg mice that received cell line 51 (Tg^+^ AT) had significantly lower bacterial burden (~4 log) in the kidneys compared with either hCD1Tg mice that only received PBS (Tg^+^ no AT) or Tg^-^ mice that had also received cell line 51 (Tg^-^ AT) (Fig 6A). Consistent with this, the Tg^+^AT group had significantly fewer infiltrating immune cells in the kidney than the other groups (Fig 6B), possibly due to lower bacterial burdens in the organ. Significantly, the adoptively transferred cell line 51 (identified using the labeling reagent CellTrace) were found in greater quantity in the livers of hCD1Tg mice than in Tg^-^ recipient mice (Fig 6C and 6D), suggesting the presence of group 1 CD1 molecules is crucial for their activation in vivo. In addition, conventional CD4^+^ T cells in the Tg^+^ AT group produced significantly more IFN-γ than the other two groups, and CD8 T cells were similarly more activated in the lymph nodes than the other groups (Fig 6E and 6F). This data suggests that activation of CD1b-restricted SA lipid-specific T cells can lead to conventional T cell transactivation during SA infection.

### SA associates with group 1 CD1-expressing APCs in vivo and early endosomes subcellularly in vitro

**Fig 7:**
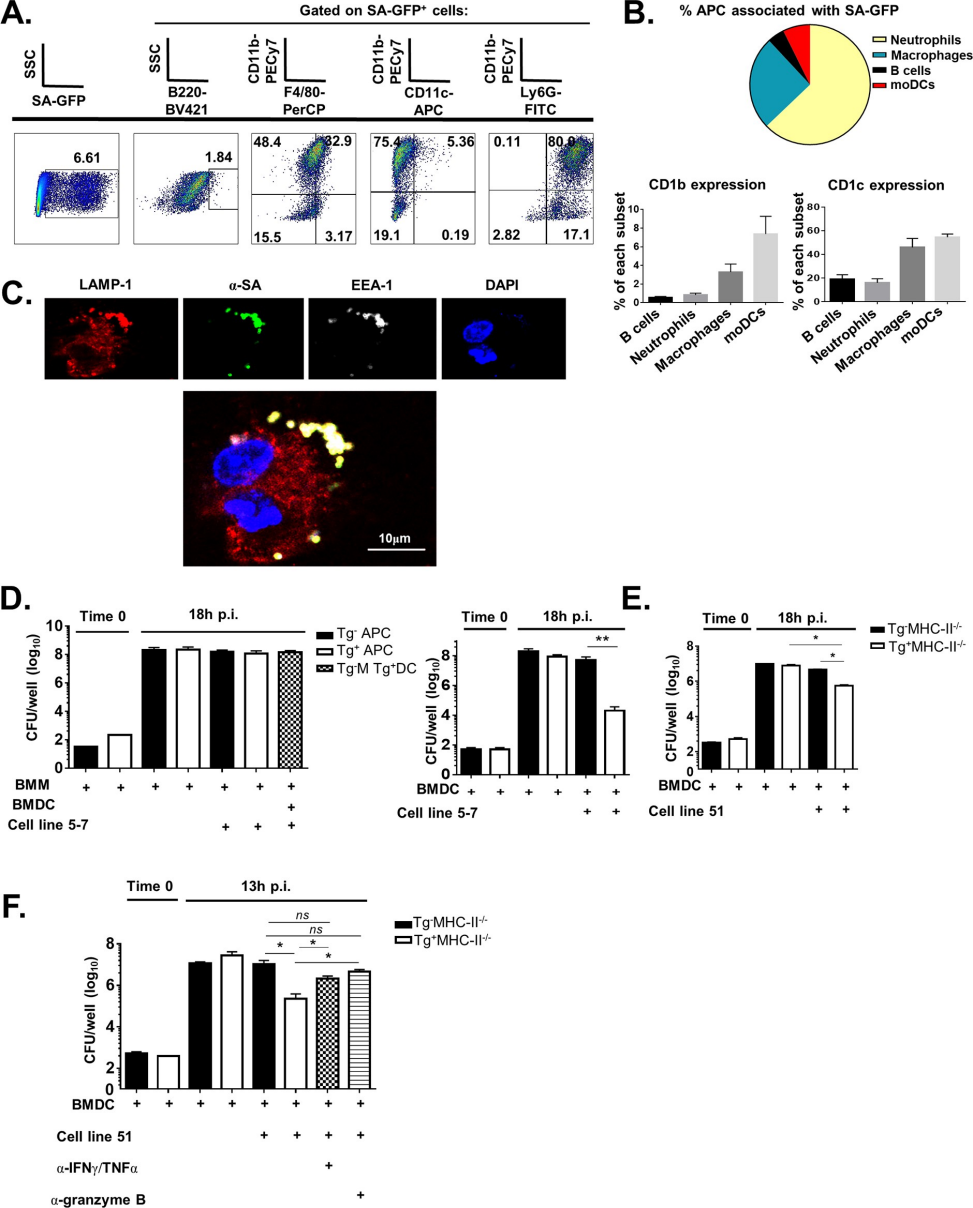
T cells specific for SA lipids help control SA growth in vitro via direct lipid antigen presentation. **(**A) hCD1Tg mice were infected with SA-GFP via tail vein. Lymphocytes from kidneys were isolated and evaluated for SA association with different APC subsets by flow cytometry. (B) Top: APC subsets associated with SA-GFP. Bottom: Quantification of APC subsets expressing CD1b or CD1c. (C) Immunofluorescent images of BMDCs 45min after internalization of SA in vitro. Cells were stained with antibodies against the endosomal markers EEA1 and LAMP1 as well as with anti-SA antibody. (D) BMMs and BMDCs infected with SA at MOI = 0.2. T cell line 5–7 cells were added to the cultures after gentamicin treatment to kill extracellular SA. Infection proceeded for 18h. Left: Only macrophages were initially infected with SA; right: only BMDCs were initially infected with SA. (E) BMDCs were infected with SA and T cell line 51 was added as above. Infection proceeded for 18h. Data representative of 3 independent experiments with all conditions plated in triplicate. (F) BMDCs were infected as above and T cell line 51 cells were added in the presence and absence of antibodies against IFN-γ and TNF-α or Granzyme B as shown. Data representative of 1 independent experiment with all conditions plated in 4x. *p<0.05, **p<0.01 using 1-way ANOVA with Bonferroni’s posttest or Student’s t test.

Protective efficacy in vivo led us to question how group 1 CD1-restricted T cells could help to kill SA in vitro. To investigate this issue, we first determined which group 1 CD1-expressing antigen-presenting cells associated with SA in vivo. hCD1Tg mice were infected with USA300 SA-GFP i.v. Mice were sacrificed at 10 days post-infection and kidney lymphocytes and kidney-draining lymph nodes were isolated and processed to determine SA co-localization with APCs. Though SA-GFP mainly associated with neutrophils (here gated on CD11b^+^Ly-6G^hi^ cells), these cells did not express high levels of CD1b or CD1c (Fig 7A and 7B). Rather, DCs and macrophages (gated here as CD11b^hi^CD11c^hi^ or CD11b^hi^F4/80^+^, respectively) comprised the majority of CD1b- and CD1c-expressing cells associated with SA-GFP (Fig 7B). We further determined the subcellular localization of SA through immunofluorescence confocal microscopy. Tg^+^ BMDCs were infected with SA and stained with antibodies against LAMP-1 (lysosomal marker), EEA-1 (marker for early endosomes), and SA. SA co-localized with LAMP-1 and EEA-1 in areas close to the cell membrane (Fig 7C), consistent with published reports that SA can survive intracellularly in membrane-associated vacuoles of human monocyte-derived macrophages [47] and is not affected by endolysosomal acidification [48]. Loading of lipid antigens onto CD1b molecules also occurs in endolysosomal compartments [49], allowing for CD1b interaction with SA lipids after internalization of SA by the cell. Uninfected BMDCs did not show EEA1-LAMP1 colocalization (S10 Fig).

### Group 1 CD1-restricted T cells can help control of SA growth in vitro

As SA primarily associated with group 1 CD1-expressing DCs and macrophages in vivo, we used these two cell types as APCs for in vitro SA infection. Tg^-^ and Tg^+^MHC-II^-/-^ BMDCs and BMMs were infected with SA in vitro. APCs lacking MHC-II were used in order to eliminate potential complications generated from superantigen and MHC-II-mediated T cell activation, and gentamicin was added 30 minutes post-infection to kill extracellular bacteria. Despite similar infection efficiencies at time 0, at 18h post-infection only the co-culture comprised of infected Tg^+^ BMDC and T cell line 5–7 was able to efficiently control SA replication by 10^4^-fold (Fig 7D, right). This demonstrated that direct Ag presentation by Tg^+^ BMDC to cell line 5–7 was required to limit SA replication in vitro. In contrast, cross-presentation of SA lipids from infected Tg^-^ macrophages incubated with Tg^+^ BMDCs to cell line 5–7 was unable to control SA replication (Fig 7D, left, hatched bar). Similarly, cell line 51 was able to control SA replication through direct Tg^+^DC-T cell contact, though to a lesser extent than cell line 5–7 (Fig 7E). Finally, antibody blockade of IFN-γ and TNF-α together or Granzyme B alone was able to reverse the protection conferred by T cell line 51 during in vitro SA infection (Fig 7F). Blocking IFN-γ or TNF-α individually did not affect the ability of group 1 CD1-restricted T cells to control SA replication in culture (S11 Fig), suggesting some redundancy of these cytokines in vitro. This demonstrates the mechanism by which group 1 CD1-restricted T cells may confer protection during SA infection.

## Discussion

The findings herein are the first to demonstrate that group 1 CD1-restricted T cells are activated by SA lipids during in vitro and in vivo MRSA infection. The absence of group 2 CD1-restricted NKT cells did not affect the magnitude or kinetics of the group 1 response to systemic SA infection. Activation of the group 1 CD1-restricted T cell response was protective against SA-induced kidney pathology, and adoptive transfer of SA lipid-specific group 1 CD1-restricted T cells into infected hCD1Tg mice significantly decreased kidney SA burdens. We additionally identified multiple SA lipid species recognized by group 1 CD1-restricted T cells during systemic SA infection. Together, our study sheds light on a non-conventional lipid-reactive T cell subset that contributes to control of MRSA infection and may aid in future vaccine design to combat MRSA.

Naïve hCD1Tg mice immunized with SA lipid-pulsed BMDCs showed robust group 1 CD1-restricted T cell responses in both lymph nodes and spleen (Fig 1). Moving forward from this proof of principle experiment, primary systemic infection with SA similarly activated group 1 CD1-restricted T cells in the lymph nodes. Intriguingly, negating the effects of superantigens in SA by infecting hCD1Tg MHC-II^-/-^ mice did not affect the magnitude of the group 1 CD1-restricted SA lipid-specific response compared with hCD1Tg WT mice, suggesting that CD4^+^ conventional T cells do not significantly contribute to background IFN-γ or IL-17 secretion when exposed to SA lipids in vivo (Fig 2B). We also addressed whether group 2 CD1-restricted T cells, or NKT cells, affected the magnitude of the group 1 CD1-restricted T cell response to SA lipids because NKT cells can also respond to a variety of microbial lipids including phosphatidyl glycerols and galactosyl diacylglycerols from different bacterial species [27, 50]. However, we found that the group 1 CD1-restricted T cell response to SA lipid was unaffected by the absence of NKT cells (Fig 2C). This is possibly because NKT cells are pre-activated innate-like lymphocytes that function early during bacterial infection [51], whereas the 10 day peak of the group 1 CD1-restricted T cell response more resembles that of conventional T cells during primary SA infection (Fig 3A, S3 Fig).

Interestingly, hCD1Tg mice showed markedly decreased kidney inflammation and pathology compared with Tg^-^WT littermate control mice (Fig 3D), despite no significant differences in CFU (Fig 3C). Immunofluorescence staining showed that SA and neutrophilic infiltrates were generally contained to small circular areas in hCD1Tg kidneys, whereas large, disordered areas of neutrophils and SA were deposited in Tg^-^WT kidneys (Fig 3E and S4 Fig). Previous studies have demonstrated that IFN-γ/IL-17^-/-^ mice develop spontaneous oral abscesses attributable to MRSA infection by 6–10 weeks of age due to dysregulated neutrophil and macrophage function [52]. SA-infected IFN-γ^-/-^ mice also displayed neutrophilic abscesses without defined borders at 3 days post-infection compared with WT mice that had clearly defined abscesses [53]. Thus, it is possible that group 1 CD1-restricted T cell production of IFN-γ and IL-17 in the lymph nodes in response to SA lipid can organize abscess formation downstream in the kidneys by affecting neutrophil recruitment and deposition.

Unbiased lipid fractionation and screening revealed that cardiolipins and polar phospholipids, specifically phosphatidyl glycerol (PG) species, are immunodominant SA lipids in the polyclonal infection setting (Fig 4B). SA lipid species, unlike the extensively characterized, relatively complex group 1 CD1 Mtb lipid antigens [54], are somewhat similar to mammalian lipids [41, 45]. However, lysyl-PG is one SA lipid component that is unique to bacteria and enriched in the immunodominant fraction 8 [55]. We initially hypothesized that lysyl-PG was responsible for the majority of IL-17 and IFN-γ secretion from group 1 CD1-restricted T cells. In addition to being a uniquely bacterial lipid, lysyl-PG also enables antibiotic resistance and immune evasion [56, 57], thus leading us to speculate that group 1 CD1-restricted T cells might aid in counteracting this mechanism in SA. Contrary to this hypothesis, when lipid antigens from the lysyl-PG-deficient *fmtC*::*erm* strain of SA were used, Fr8 retained the ability to activate group 1 CD1-restricted T cells (Fig 4D), implicating PG and not lysyl-PG species as immunodominant SA lipids. PG is known to be a CD1b-restricted T cell antigen; one previous study identified an autoreactive CD1b-restricted T cell clone from healthy human donors not known to be SA-infected that could cross-react with PG isolated from *S*. *aureus* and synthetic mammalian 18:0/18:1 PG [39]. This appears to differ from our results in the polyclonal setting in that hCD1Tg mice infected with SA displayed robust IL-17 secretion from group 1 CD1-restricted T cells in response to PG-rich Fr8 from SA but not to synthetic mammalian 18:0 PG (S6 Fig). Thus we hypothesize that a subset of SA PG-specific, non-autoreactive group 1 CD1-restricted T cells are induced during SA infection. SA-derived cardiolipins also activated group 1 CD1-restricted T cells in the polyclonal SA infection setting (Fig 4A). Though much larger than phosphatidyl glycerol moieties also presented by CD1b molecules[39], we suspect that CD1b has a large enough binding groove to accommodate cardiolipin species. Indeed, CD1b presents Mtb lipids with acyl chain lengths that are 80 carbons long [36]; thus we anticipate CD1b would also be able to bind and present cardiolipin. Intriguingly, the cardiolipin-enriched Fr3 selectively induced IL-17 but not IFN-γ production from group 1 CD1-restricted T cells (Fig 4B). This may be attributable to the binding affinity of cardiolipin-CD1 engagement with the group 1 CD1-restricted TCR, as has been shown in CD4^+^ conventional T cells to promote T_h_17 differentiation [58]. It is also possible that the group 1 CD1-independent IFN-γ production observed in Fr3-stimulated lymphocytes was mediated through TCR-independent, cytokine-driven mechanisms. The studies contained herein are the first to our knowledge to identify SA-derived cardiolipins as group 1 CD1-binding lipid antigens, though further study using cell-free target systems is needed to verify TCR dependence in these responses.

SA and gram-positive bacteria in general contain a diversity of PG species that elute differently according to the solvent gradient. It is interesting to note that not all PG-rich fractions of SA lipids induced group 1 CD1-dependent T cell secretion of cytokine (Fig 4B). A comprehensive LC-Q-TOF-MS analysis of SA lipids demonstrated that PG species of different chain lengths eluted at different times in HPLC [41], and a recent study on *Enterococcus faecalis* lipids showed similar findings [59]. Indeed, silica column fractionation of total SA lipids resulted in multiple fractions enriched with different PG species depending on the ratio of CHCl_3_:MeOH used (Fig 4A). It is possible that though PG species in SA contain the same polar headgroups, the differences in fatty acyl chain lengths between PG moieties may affect binding affinity to the group 1 CD1 antigenic groove and subsequent TCR engagement strength. Previous studies have shown that CD1b binds diverse sulfoglycolipids from Mtb with the same polar headgroup but differing acyl chain lengths, which results in altered TCR recognition and T cell activation [38]. A similar mechanism could be at play regarding PG moieties from SA. Additionally, it is known that variations in the branching patterns of alkyl chains can also affect lipid Ag binding onto CD1c and downstream T cell activation [60]. Indeed, SA contains an abundance of anteiso- and iso-branched fatty acyl chains associated with PG species in the bacterial cytoplasmic membrane [61] that are not present in mammals, which may uniquely activate group 1 CD1-restricted T cells. Further characterization by GC-MS or ESI-tandem mass spectrometry is needed to determine if these subtle structural differences exist between SA PG species, and whether they contribute to immunodominance hierarchies in the polyclonal infectious setting.

Recent studies have shown that T cell production of IL-17 and IFN-γ is essential for protection against SA in both mice and humans [16, 18, 19, 53]. Despite this, vaccine trials focused on activating CD4^+^ conventional T cell responses specifically have largely failed in humans [13, 62]. It is therefore important to examine whether activation of non-conventional T cells during SA infection can be protective. Adoptive transfer of the group 1 CD1-restricted SA lipid-specific T cell line CTL51 decreased SA kidney burdens by ~10^4^ fold at 4 days post-infection in hCD1Tg but not Tg^-^WT mice (Fig 6A). This effect was correlated with trans-activation of CD4 and CD8 T cells in the draining lymph nodes (Fig 6E and 6F), consistent with where the group 1 CD1-restricted T cell response occurred during primary SA infection (Fig 2). The magnitude of protection afforded by the adoptively transferred SA-lipid-specific group 1 CD1-restricted T cells suggests that it will be important to elucidate how best to activate group 1 CD1-restricted T cell responses during infection, as this could provide a robust, novel protective mechanism against systemic SA infection.

Though primarily considered an extracellular pathogen, studies have shown that SA can enter into macrophages, DCs, neutrophils, and even non-phagocytic cells [63]. Our data show that SA is primarily associated with neutrophils and macrophages, and to a lesser extend with DCs in the kidney (Fig 7A and 7B). While CD1b and CD1c are highly expressed on a subset of myeloid-derived dendritic cells, neutrophils and macrophages do not express CD1b and neutrophils express only low levels of CD1c. This provided the justification for the use of macrophages and DCs for in vitro infection experiments. The co-culture of group 1 CD1-restricted T cells with SA-infected hCD1Tg^-^ and Tg^+^ APCs demonstrated a critical role for direct SA lipid presentation by group 1 CD1-expressing DCs to group 1 CD1-restricted T cells in controlling SA growth (Fig 7D). Indeed, no control of SA infection was observed when infected BMM were co-incubated with SA lipid-specific T cells with uninfected Tg^+^ BMDC help, suggesting that cross-presentation of SA lipids to Tg^+^ DCs is not effective in halting SA replication. In contrast, control of SA in culture was orders of magnitude higher when infected Tg^+^ DCs directly presented lipids to T cell line 5–7 (Fig 7D, bottom panel). One possible explanation for this is that BMM differentiated in culture do not express CD1b [33], and our group 1 CD1-restricted T cell lines are generally CD1b-restricted (Fig 5E). Though cell line 5–7 is both CD1b and CD1c-restricted (Fig 5E), the binding affinity of CD1b loaded with SA lipid to the cell line 5–7 TCR may be higher than the CD1c-TCR binding affinity, allowing for differential cytokine producing capacities during in vitro SA infection. This is still purely speculative, as one limitation of our study was that we could only indirectly show TCR dependence for activation in our group 1 CD1-restricted cell lines (S9 Fig). Another possibility is that SA internalized by BMM can easily escape mature phagolysosomes and kill the cells [48], which would limit TCR engagement. Dendritic cell cross presentation of SA lipids from infected macrophages may also be less efficient at activating group 1 CD1-restricted T cells because the time spent internalizing apoptotic macrophages and processing/presenting SA lipids may take too long, allowing bacteria to overgrow in the culture. Additionally, BMDCs may be more resistant to killing by SA, as one study examining DC-*S*. *aureus* interactions in vitro showed efficient control of infection of BMDCs out to 18h post-infection [64]. This would allow more time for group 1 CD1-restricted T cell activation in culture.

Finally, blockade of either TNF-α and IFN-γ together or granzyme B alone was sufficient to limit the protective efficacy of group 1 CD1-restricted T cell line 51 (Fig 7F). T cell secretion of granzyme B protects against bacterial growth in a variety of infection models. Enhanced clearance of pulmonary MRSA was observed in mice with elevated granzyme B levels, a phenotype which was replicated in human neutrophil culture [65]. Though this phenotype depended on neutrophil secretion of granzyme B, it is likely that T cell production of granzyme B has similar effects. Granzyme B production from a CD4^+^ human T cell clone also protected against Mtb growth in macrophages in vitro [66], and resistance to Mtb infection in humans is correlated with elevated MAIT cell production of granzyme B [67]. Similarly, intracellular growth of *Listeria monocytogenes* (LM) within HeLa cells and monocyte-derived dendritic cells was inhibited by human LM-specific CTL in a granzyme B-dependent manner [68], suggesting that granzyme B is broadly effective in killing multiple species of intracellular bacteria. Similarly, studies have shown that both IFN-γ and TNF-α contribute to host resistance against systemic SA infection [69], and that γδ T cell production of both cytokines controlled SA replication in a mouse skin infection model [70]. Though blocking either IFN-γ or TNF-α individually in vitro did not affect SA replication compared to the Tg^+^ DC-T cell condition (S11 Fig), blocking both simultaneously enhanced replication (Fig 7F). This suggests that IFN-γ and TNF-α production by group 1 CD1-restricted T cells in vitro play a redundant role in controlling SA replication.

*Staphylococcus aureus* has proven to be a stubborn pathogen to eradicate or develop vaccines against. This highlights the importance of elucidating novel pathways of T cell activation during SA infection to expand the parameters of vaccine design. The studies herein are the first to our knowledge to demonstrate that lipid-specific group 1 CD1-restricted T cells can be activated during systemic SA infection. Notably, these T cells primarily respond to SA-derived phospholipids and can control bacterial growth both in vitro and in vivo settings. These data suggest that incorporating SA lipid antigens into a multi-component subunit vaccine which targets both unconventional and conventional T cells may be effective in helping combat this complex public health crisis.

## Materials and methods

### Ethics statement

This study was carried out in strict accordance with the recommendations in the Guide for the Care and Use of Laboratory Animals of the National Institutes of Health. The protocol was approved by the Animal Care and Use Committee of the Northwestern University (Protocol number: IS00001659).

### Mouse strains

hCD1Tg mice (Line 78 and Line 64) in the B6 background were generated in our lab as previously described [32]. hCD1Tg mice were also crossed onto B6 MHC-II-deficient or CD1d-deficient background [71]. Most in vivo infection experiments used hCD1Tg (Line 78) mice expressing CD1b and CD1c but not CD1a due to better breeding performance. Proof of principle experiments used hCD1Tg (Line 64) mice expressing CD1a, CD1b and CD1c in a tissue-specific, physiologically relevant manner.

### Bacterial strains, media, and culture conditions

Mouse intravenous infections with *Staphylococcus aureus* (SA) were carried out using the methicillin resistant USA300 strain LAC or LAC-GFP [72]. SA was grown shaking overnight in TSA broth (MP Biomedicals, Solon, OH) at 37°C, 220 rpm. A marked fmtC::erm insertional disruption in strain LAC was generated by bacteriophage-mediated transduction using ϕ11 containing DNA from transposon mutant NE1360 of the Nebraska transposon mutant library [73]. SA lipids were isolated from either WT LAC or *fmtC*::*erm* LAC.

### Primary cell preparation

Single cell suspensions were prepared from livers, spleens, pooled peripheral lymph nodes, and/or kidneys of infected or immunized hCD1Tg mice. Lymphocytes were isolated from livers and kidneys using a 37.5% Percoll gradient centrifugation. At times, αβ T cells were enriched through depletion of B220^+^, CD11c^+^, F4/80^+^, Ly-6G^+^, MHC-II^+^, and TCRγδ+ T cells using biotin-conjugated antibodies (BioLegend, San Diego, CA) and the Dynabeads depletion kit (ThermoFisher Scientific, Waltham, MA). BMDCs were derived from mouse bone marrow progenitors using GM-CSF and IL-4 (PeproTech) as previously described [74]. BMM were derived from bone marrow progenitors using complete DMEM supplemented with 30% L929-conditioned medium or 20ng/mL recombinant MCSF (PeproTech).

### Antibodies and flow cytometry

CD1a-PE, CD1b-PE, CD1c-FITC, B220-PerCPCy5.5, F4/80-APC, CD11c- BV421, CD8-BV510, CD4-PerCPCy5.5, IFN-γ-APC, IL-17-PE, Granzyme B-BV421, and TNF-α-FITC antibodies were purchased from BioLegend (San Diego, CA). TCR-β-BV421, anti-Vβ3 and 4-PE, and anti-Vβ5.1/5.2-APC were obtained from BD biosciences (San Jose, CA). For flow cytometry, single cell suspensions from organs were incubated with 2.4G2 FcR blocking antibody for 10 minutes and then stained in HBSS-2% FBS containing 50 μg/mL gentamicin. Samples were run on the FACS Canto II flow cytometer (BD Biosciences, San Jose, CA). Analysis of flow cytometry data was performed on FlowJo software (Tree Star, Inc). S12 Fig shows the gating strategies employed for the FACS plots of group 1 CD1-restricted T cells in the polyclonal setting in Fig 3 and group 1 CD1-restricted T cell line functional assays in Fig 5.

### Lipid isolation from Staphylococcus aureus

USA300 SA-derived total lipid was isolated from overnight cultures of USA300 WT or *fmtC*::*erm* grown in TSB, shaking at 220rpm, 37°C. Bacteria were pelleted, washed in PBS, and resuspended in 50 nM KH_2_PO_4_ buffer containing 2 μg/mL lysostaphin in order to lyse the peptidoglycan cell wall and release protoplasts. Bacteria pellet were washed with PBS and then resuspended in 100% EtOH for further processing. Total SA lipids were isolated from protoplasts using a CHCl_3_-MeOH Bligh-Dyer extraction procedure [75]. Sub-fractionation was carried out using silica gel column chromatography (0.5 x 20cm) using a stepwise elution procedure with chloroform, acetone, and methanol in chloroform as solvents (15mL per fraction). Lipids were characterized by 1D- and 2D-TLC using hexane/diethyl ether/acetic acid (85:15:1, v/v) for 1-D TLC and chloroform/methanol/28% ammonia/toluene (64:30:6:10, v/v) as solvent A and chloroform/methanol/acetone/acetic acid/water/toluene (70:30:5:4:1:10, v/v) as solvent B. Lipid visualization was performed with 10% sulfuric acid in methanol with heating or using phosphomolybdate reagent to detect phospholipids. [76]. Lipids were also characterized by electrospray ionization tandem mass spectrometry (ESI-MS/MS) using the AB Sciex 6500 QTRAP mass spectrometer.

### Intravenous SA infection and DC immunization of mice

*S*. *aureus* overnight cultures were diluted 1:100 and subsequently grown to mid-log phase (~3 hours) shaking at 37°C, 220 rpm. Bacteria were washed in PBS and mice were infected with 3-5x10^6^ CFU in sterile PBS via tail vein injection. At indicated times, lymphocytes isolated from various organs were harvested and analyzed by flow cytometry and ELISPOT assays. Kidney homogenates from infected mice were plated on tryptic soy agar (TSA) plates to determine viable CFU. For immunization, hCD1Tg MHC-II^-/-^ DCs were pulsed overnight with total SA lipids (10 μg/mL). 10^6^ BMDCs were injected intraperitoneally into mice and spleens and lymph nodes isolated for ELISPOT assays 7 days post-immunization.

### ELISPOT assays

Multiscreen-IP plates (Millipore-Sigma) were coated with 5 μg/mL anti-IFN-γ (clone AN-18, BioLegend) or anti-IL-17 (clone eBioCK15A5, Thermo Scientific), washed, and blocked with RPMI-10. Lymphocytes from spleens or pooled peripheral lymph nodes from immunized or infected mice were added to each well containing 5x10^4^ hCD1Tg^-^ or Tg^+^MHC-II^-/-^ BMDCs pulsed with total SA lipids or lipid fractions, purified mammalian 16:0–18:1 cardiolipin, mammalian 18:0 phosphatidyl glycerol, or bacteria-derived 16:0 lysyl PG (Avanti Polar lipids, Alabaster, AB). Plates were incubated at 37°C for 20 h and developed using an Alkaline Phosphatase Conjugate Substrate Kit according to manufacturer’s instructions (Bio-Rad Laboratories, Carlsbad, CA). IFN-γ or IL-17-producing cells were quantified using an ImmunoSpot reader (Cellular Technologies, Ltd., Shaker Heights, OH).

### Generation of group 1 CD1-restricted T cell lines

Lymphocytes isolated from SA-infected hCD1Tg mice were cultured for 4 days in RPMI-10 and subsequently in supplemented Eagle’s minimum essential medium with 10U/mL recombinant mouse IL-2 (PeproTech, Rocky Hill, NJ) [77]. T cell lines were re-stimulated weekly with irradiated SA lipid pulsed hCD1Tg MHC-II^-/-^ BMDCs. T cell clones 6, 51, and 5–7 were generated via limiting dilution cloning.

### Adoptive transfer of group 1 CD1-restricted T cell lines

T cell line 51 was labeled with CellTrace Violet (Thermo Scientific) dye prior to injection into mice. 2x10^6^ cells in 200 μL of PBS were injected into hCD1Tg or Tg^-^ sex-matched mice via tail vein 1 day prior to infection. Control hCD1Tg mice received PBS alone. All mice were infected with 2x10^6^ CFU of SA USA300 via tail vein at day 0 and euthanized at 4 days post-infection. Livers, spleens, peripheral lymph nodes and kidneys were isolated for CFU enumeration or functional experiments. Lymphocytes from these organs were stimulated with phorbol myristate acetate and ionomycin (PMA-Iono) at a concentration of 20 ng/mL and 1μM, respectively, for 6 h. Brefeldin A (10 μg/mL) was added to the cells at 2 h post-stimulation to detect intracellular cytokine production.

### In vitro infections

BMM (5x10^4^/well) and BMDC (10^5^ cells/well) were plated in triplicate in 48 well plates and infected with SA USA300 at MOI = 0.2. After 30 min, the medium was replaced with DMEM containing 100μg/mL gentamicin to kill extracellular bacteria for 30 minutes. Cells were then washed twice in warm PBS and a portion of BMM and BMDC were lysed in the wells with 300 μL sterile H_2_O to plate intracellular bacteria for Time 0 CFU enumeration. The remainder of the cells received either media alone or had uninfected BMDCs (10^5^/well), BMMs (5x10^4^/well), or T cells (4x10^5^/well) added to the wells. Some experiments included antibody blockade of cytokine production; 1μg/well was added of each of the following: α-IFN-γ (cloneAN-18), α-TNF-α (clone 6B8), or α-Granzyme B (clone QA16A02) (all from BioLegend). For antibody blocking experiments, all conditions were plated in groups of 4. Cells were spun down to enable contact and incubated for 18 hours at 37°C. Plates were then spun down at 2400 rpm x 15 minutes to pellet SA, supernatant removed, and cells lysed in the wells with 300 μL sterile H_2_O. Samples were serially diluted and spot-plated for CFU enumeration on TSA plates containing 5 μg/mL erythromycin.

### Immunofluorescence on APCs

BMDCs and BMMs (10^6^ cells) were plated onto glass coverslips coated with poly-L-lysine inside 12-well plates and allowed to adhere for 1 hour. Cells were left uninfected or were infected with SA at MOI = 5 for 45 minutes. Cells were then washed twice with PBS and resuspended in DMEM containing 100 μg/mL gentamicin to kill extracellular bacteria for 30 minutes at 37°C. Cells were washed, fixed with 10% PBS-buffered formalin containing zinc, and stained with the following primary antibodies in PBS + 1% BSA: polyclonal rabbit anti-mouse EEA-1, rat anti-mouse LAMP1 (clone 1D4B), and mouse anti-Staphylococcus aureus (clone 704) from Abcam (Cambridge, MA). Alexa fluor-conjugated secondary antibodies were goat anti-mouse IgG H&L-AF488 (Abcam), goat anti-rat IgG H&L-AF568 and goat anti-rabbit IgG H&L-AF647 (both from ThermoFisher, Waltham, MA). Cells were incubated in Vectashield + DAPI and placed onto glass slides for imaging. Microscopy was performed using a confocal Nikon A1R (A) spectral microscope (Nikon, Melville, NY).

### Immunofluorescence on paraffin-embedded sections

Immunofluorescence on kidneys was performed as previously described [78]. Briefly, paraffin-embedded kidneys deparaffinized with xylene and rehydrated with graded ethanol washes. Antigen retrieval was performed using sodium citrate buffer in a pressure cooker. Endogenous peroxidases were quenched with 10% H_2_O_2_. Background Buster blocking solution (Innovex Biosciences, Richmond, CA) was applied to each sample prior to incubation in primary antibody. Slides were then incubated sequentially with rabbit polyclonal anti-SA (Abcam) and rat anti-mouse Ly-6G (clone 1A8) for 1 hour at room temperature or overnight at 4°C. Slides were then incubated sequentially with goat anti-rat AF-555 (Invitrogen) or goat anti-rabbit AF488 (Abcam) for 1 hour at room temperature. Slides were then mounted using Vectashield Antifade Reagent with DAPI (Vector Labs). Slides were imaged at 25°C, 20x magnification, using the Nikon A1 HD25 confocal microscope (Nikon Instruments Inc, Melville, NY). Images were analyzed with NIS Elements Viewer 4.20 software.

### Statistical analysis

Statistical analyses were performed using Prism software (GraphPad Software, Inc., San Diego, CA). Significant differences at 95% confidence or above are depicted with asterisks on each graph.

## Supporting information

S1 Fig SA lipid-specific group 1 CD1-restricted T cell responses can be detected in hCD1Tg mice expressing CD1a, -b, and –c during systemic SA infection. hCD1Tg mice expressing CD1a, -b, and -c (Tg64) were infected with 5x10^6^ CFU of SA USA300 via tail vein. Mice were sacrificed at 10 days post-infection and peripheral lymph nodes were isolated. Lymphocytes were cultured overnight with SA lipid pulsed or unpulsed Tg^-^ and Tg^+^ MHC II^-/-^ BMDCs and assayed for IL-17A production via ELISPOT. Data representative of 2 independent experiments with n = 5–7 mice per experiment. *p<0.05 using a two-tailed Student’s t test.
(TIF)

S2 Fig Group 1 CD1-restricted T cell responses against SA lipids cannot be detected in the kidney at 10 days post-infection. hCD1Tg mice were infected with 5x10^6^ CFU of USA300 via tail vein. Mice were euthanized at 10 days post-infection and lymphocytes from the kidney and associated lymph nodes were isolated and cultured with the indicated BMDC targets overnight. IFN-γ (right) and IL-17A (left) production was assessed by ELISPOT assays. Data representative of 2 independent experiments with n = 3–4 mice per experiment.
(TIF)

S3 Fig Activation kinetics of conventional T cells and the expression of group 1 CD1 during the course of SA infection. **(**A) Tg^-^WT mice were infected with 5x10^6^ CFU of USA300 i.v. and sacrificed at the indicated times post-infection. Lymphocytes from lymph nodes were stained with T cell-specific antibodies for FACS. Cells were gated on either TCRβ^+^CD4^+^NK1.1^-^ cells or TCRβ^+^CD8^+^ cells and output for CD69 expression. (B, C) hCD1Tg mice were infected as above and sacrificed at the indicated times. Lymphocytes from pooled peripheral lymph nodes were analyzed by FACS for CD1b and CD1c expression at different times post-infection. Data representative of 4 independent experiments with n = 4–5 mice per time point. *p<0.05; **p<0.01; ***p<0.005 using one-way ANOVA with Tukey’s post-test.
(TIF)

S4 Fig hCD1Tg mice display less kidney pathology than Tg^-^ WT mice in response to SA infection. hCD1Tg and Tg^-^WT littermate control mice were infected with 3x10^6^ CFU of SA via tail vein. Mice were euthanized at 10 days post-infection and kidneys were isolated and processed for H&E staining. Panels show whole kidney sections containing areas of inflammation and mature abscess formation, with Tg^-^ WT mice more affected than hCD1Tg^+^ mice. Data representative of 2 independent experiments with n = 4 mice per group.
(TIF)

S5 Fig SA lipid fractions enriched in phospholipids are heterogeneous. (A) Table showing percentage of PG species present in each PG-rich fraction classified according to acyl chain length. Cardiolipin-enriched Fraction 3 has the same chain length distribution as it is simply a dimer of PG species. (B) Lipid fractions were subjected to TLC separation using chloroform: methanol: acetone: acetic acid: water: toluene (70:30:5:4:1:10, v/v) as a solvent system. Phospholipids in each fraction (right panel) were visualized using phosphomolybdate reagent (blue spots) as described in Vaskovsky *et al*. [76]. Total lipids were visualized by “charring” or heating the same blot used to visualize phospholipids (left). Increasing concentrations of lysyl-PG is the main distinguishing characteristic between PG-enriched fractions 6, 7, and 8.
(TIF)

S6 Fig USA300 *fmtC*::*erm* mutant specifically lacks lysyl-PG but retains PG species. Mass spectra showing that the *fmtC*::*erm* mutant strain of SA (bottom panel) retains all other major SA lipid moieties except for lysyl-PG species.
(TIF)

S7 Fig Purified mammalian cardiolipin, PG and synthetic lysyl-PG cannot activate group 1 CD1-restricted T cells from SA-infected mice. hCD1Tg mice were infected with 3x10^6^ CFU of SA via tail vein. Mice were euthanized at 10 days post-infection and lymphocytes from pooled peripheral lymph nodes were put into IL-17A ELISPOT. Data representative of 2 independent experiments with n = 4 mice per experiment. *p<0.05 using two-way ANOVA with Tukey’s posttest.
(TIF)

S8 Fig SA lipids enhance BMDC activation to similar levels in Tg^-^ and Tg^+^ BMDCs, irrespective of group 1 CD1 expression. **(**A, B) Quantification of CD86 (A), CD1b, and CD1c (B) expression in unstimulated or SA lipid stimulated Tg^-^ and Tg^+^ BMDCs. (C) Tg^-^ and Tg^+^ DCs produced similar levels of IL-6 in response to SA lipids. Tg^-^ and Tg^+^ BMDCs were stimulated with the indicated SA lipids/fractions for 12h and supernatants were assayed for IL-6 production by ELISA.
(TIF)

S9 Fig MyD88-independent cytokine production by group 1 CD1-restricted SA-lipid-specific T cell lines. T cell lines 51, 6, and 5–7 were stimulated with Tg^-^ and Tg^+^ BMDCs coated with SA lipids and lacking the MyD88 adaptor protein for NFκB signaling. Cells were co-cultured overnight (T cell line 6, 51) or for 48h (T cell line 5–7) and IFN-γ ELISPOT (T cell line 6, 51) or ELISA (T cell line 5–7) was performed. Data shown is after subtracting background from Tg^-^ or Tg^+^ BMDC unstimulated conditions. Data representative of 2–3 independent experiments with each condition plated in duplicate. **p<0.01 using two-way ANOVA with Tukey’s posttest.
(TIF)

S10 Fig EEA1 and LAMP1 colocalization depends on SA infection in vitro. Immunofluorescence staining against LAMP1, EEA1, and SA was conducted on uninfected or SA-infected BMDCs. Data representative of 2 independent experiments with all conditions stained in triplicate.
(TIF)

S11 Fig Blocking of granzyme B but not TNF-α or IFN-γ individually reduce the ability of cell line 5–7 to help control SA replication in vitro. BMDCs were infected with SA as in Fig 7 and infection proceeded for 13h. Some conditions included antibody against IFN-γ or TNF-α or granzyme B in each well. Cells were lysed in the wells and bacteria pelleted and plated for CFU enumeration. All conditions were plated in triplicate. Data representative of 3 independent experiments. *p<0.05 using Student’s t test.
(TIF)

S12 Fig Gating strategies for Figs 3B and 5C. Fig 3B is gated on lymphocytes from pooled peripheral lymph nodes of hCD1Tg^+^ mice after depletion of endogenous antigen presenting cells and γδ T cells. Fig 5C is gated on lymphocytes from the primary T cell line CTL 5–7.
(TIF)
