# Supplementary figures and images for: Group 1 CD1-restricted T cells contribute to control of systemic Staphylococcus aureus infection
Source: PLoS Pathog. 2020 Apr 28;16(4):e1008443. doi: 10.1371/journal.ppat.1008443 (PMC7188215; doi:10.1371/journal.ppat.1008443)

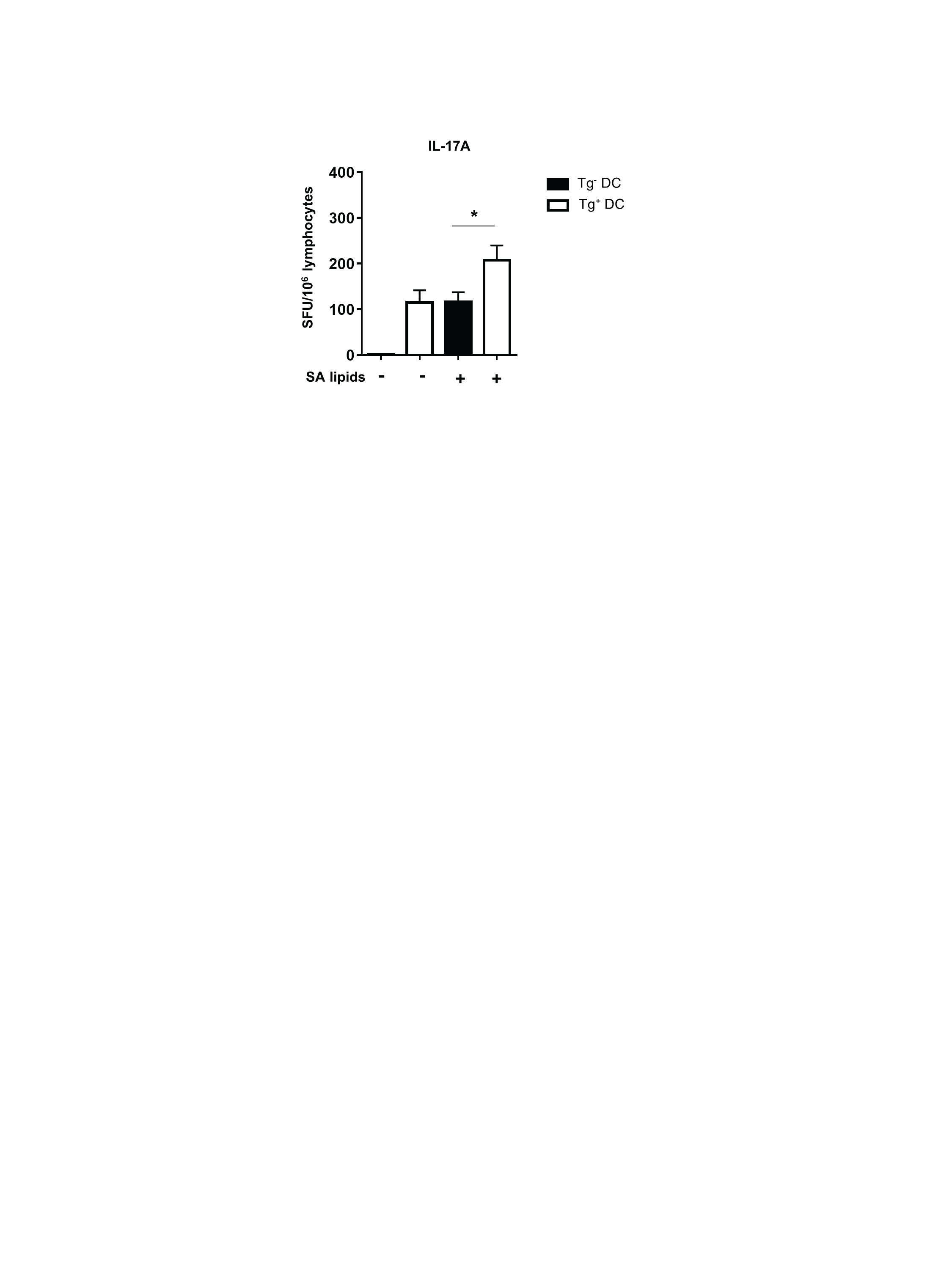

Supplement: S1 Fig — hCD1Tg mice expressing CD1a, -b, and -c (Tg64) were infected with 5x10^6^ CFU of SA USA300 via tail vein. Mice were sacrificed at 10 days post-infection and peripheral lymph nodes were isolated. Lymphocytes were cultured overnight with SA lipid pulsed or unpulsed Tg- and Tg+ MHC II-/- BMDCs and assayed for IL-17A production via ELISPOT. Data representative of 2 independent experiments with n = 5–7 mice per experiment. *p<0.05 using a two-tailed Student’s t test. (TIF) [file ppat.1008443.s001.tif]

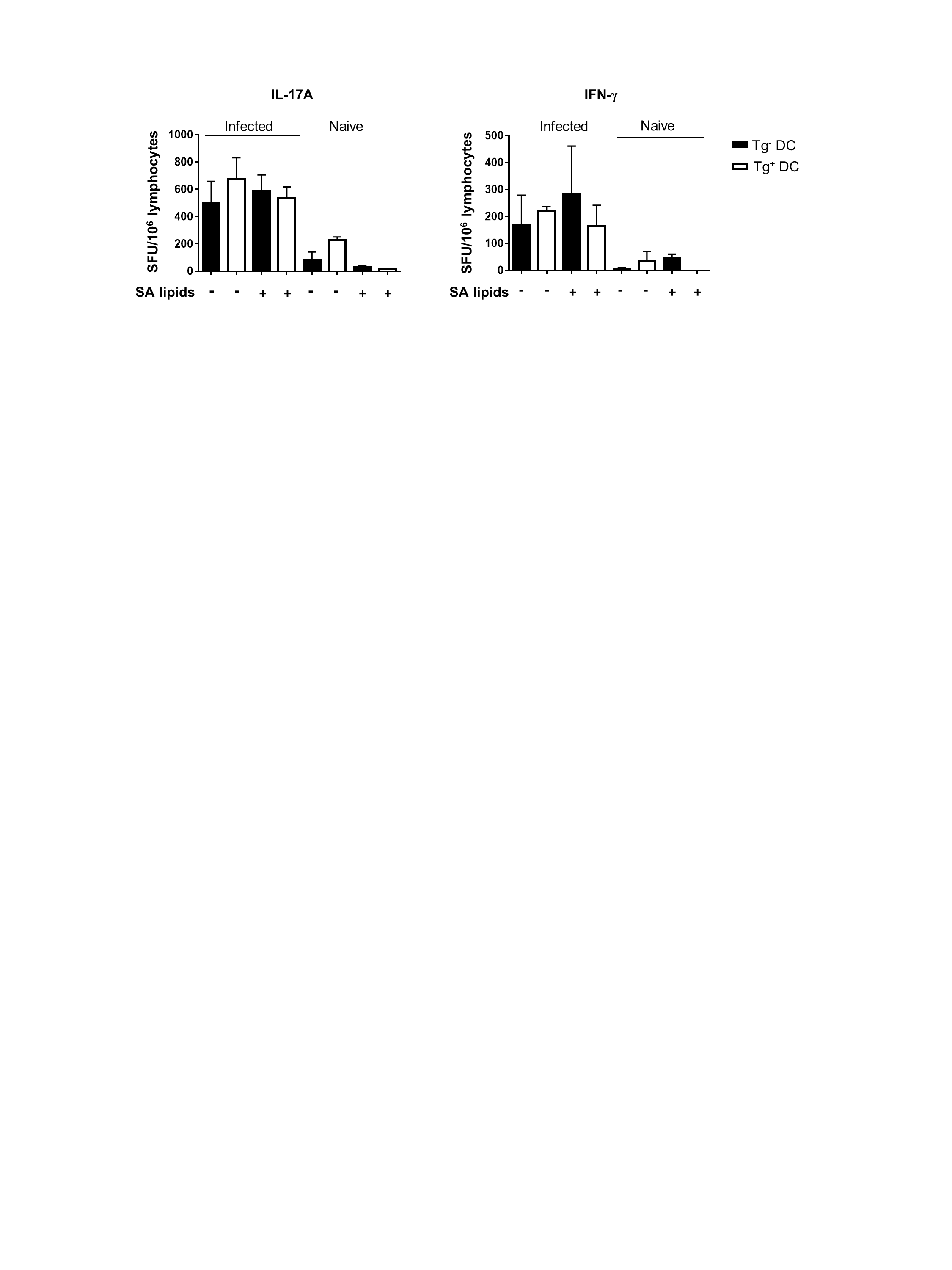

Supplement: S2 Fig — hCD1Tg mice were infected with 5x10^6^ CFU of USA300 via tail vein. Mice were euthanized at 10 days post-infection and lymphocytes from the kidney and associated lymph nodes were isolated and cultured with the indicated BMDC targets overnight. IFN-γ (right) and IL-17A (left) production was assessed by ELISPOT assays. Data representative of 2 independent experiments with n = 3–4 mice per experiment. (TIF) [file ppat.1008443.s002.tif]

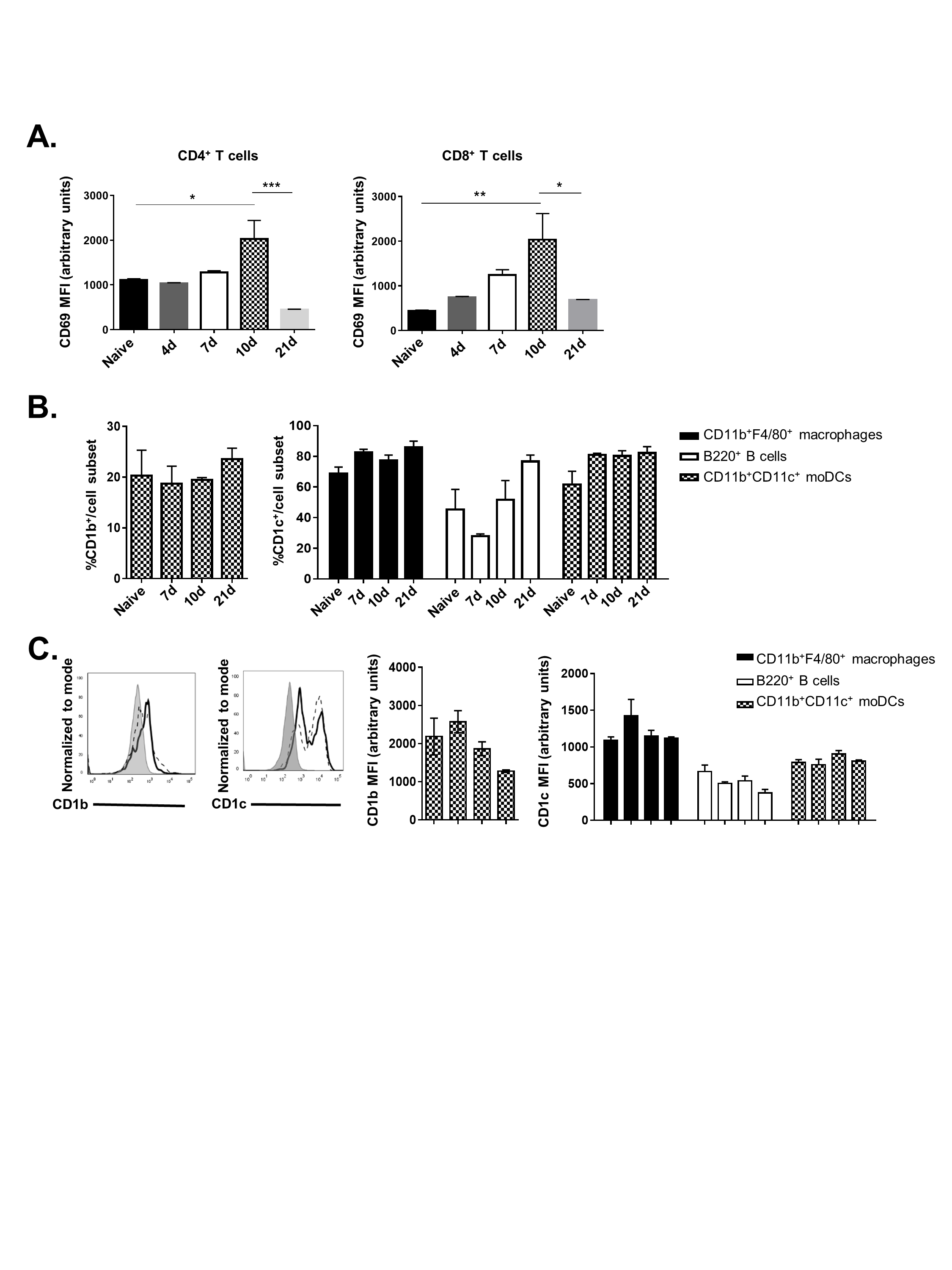

Supplement: S3 Fig — (A) Tg-WT mice were infected with 5x10^6^ CFU of USA300 i.v. and sacrificed at the indicated times post-infection. Lymphocytes from lymph nodes were stained with T cell-specific antibodies for FACS. Cells were gated on either TCRβ+CD4+NK1.1- cells or TCRβ+CD8+ cells and output for CD69 expression. (B, C) hCD1Tg mice were infected as above and sacrificed at the indicated times. Lymphocytes from pooled peripheral lymph nodes were analyzed by FACS for CD1b and CD1c expression at different times post-infection. Data representative of 4 independent experiments with n = 4–5 mice per time point. *p<0.05; **p<0.01; ***p<0.005 using one-way ANOVA with Tukey’s post-test. (TIF) [file ppat.1008443.s003.tif]

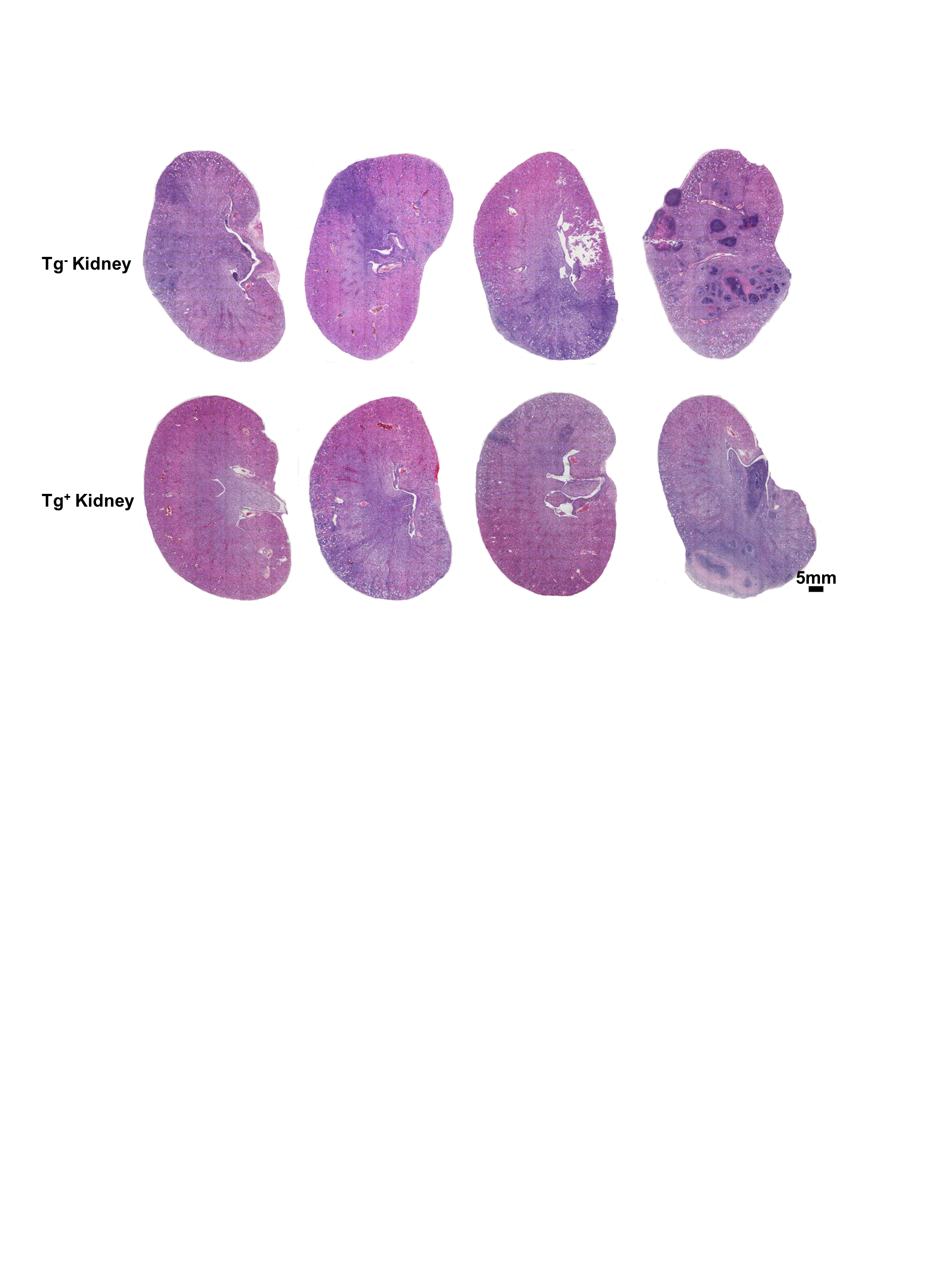

Supplement: S4 Fig — hCD1Tg and Tg-WT littermate control mice were infected with 3x10^6^ CFU of SA via tail vein. Mice were euthanized at 10 days post-infection and kidneys were isolated and processed for H&E staining. Panels show whole kidney sections containing areas of inflammation and mature abscess formation, with Tg- WT mice more affected than hCD1Tg+ mice. Data representative of 2 independent experiments with n = 4 mice per group. (TIF) [file ppat.1008443.s004.tif]

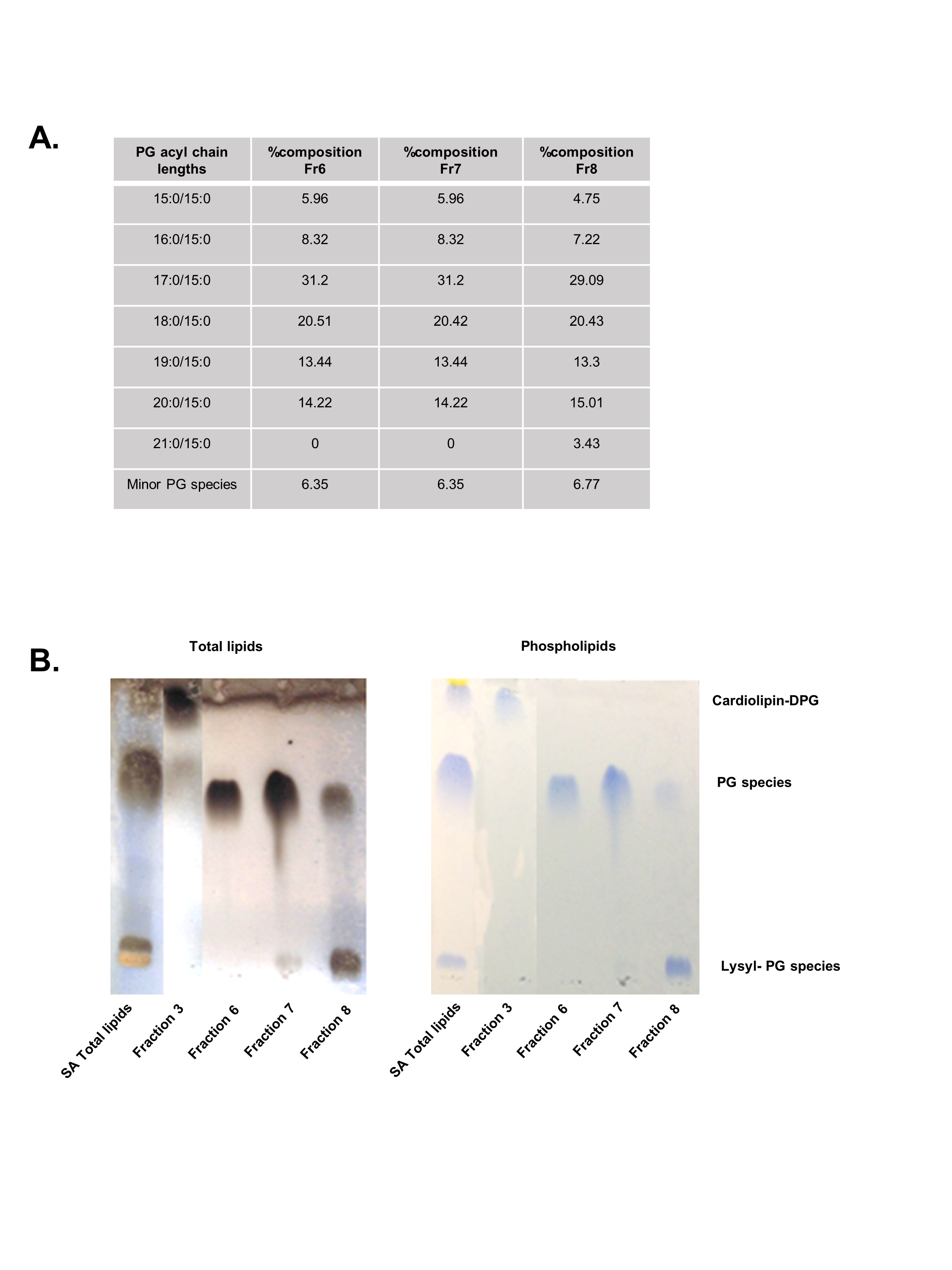

Supplement: S5 Fig — (A) Table showing percentage of PG species present in each PG-rich fraction classified according to acyl chain length. Cardiolipin-enriched Fraction 3 has the same chain length distribution as it is simply a dimer of PG species. (B) Lipid fractions were subjected to TLC separation using chloroform: methanol: acetone: acetic acid: water: toluene (70:30:5:4:1:10, v/v) as a solvent system. Phospholipids in each fraction (right panel) were visualized using phosphomolybdate reagent (blue spots) as described in Vaskovsky et al. [76]. Total lipids were visualized by “charring” or heating the same blot used to visualize phospholipids (left). Increasing concentrations of lysyl-PG is the main distinguishing characteristic between PG-enriched fractions 6, 7, and 8. (TIF) [file ppat.1008443.s005.tif]

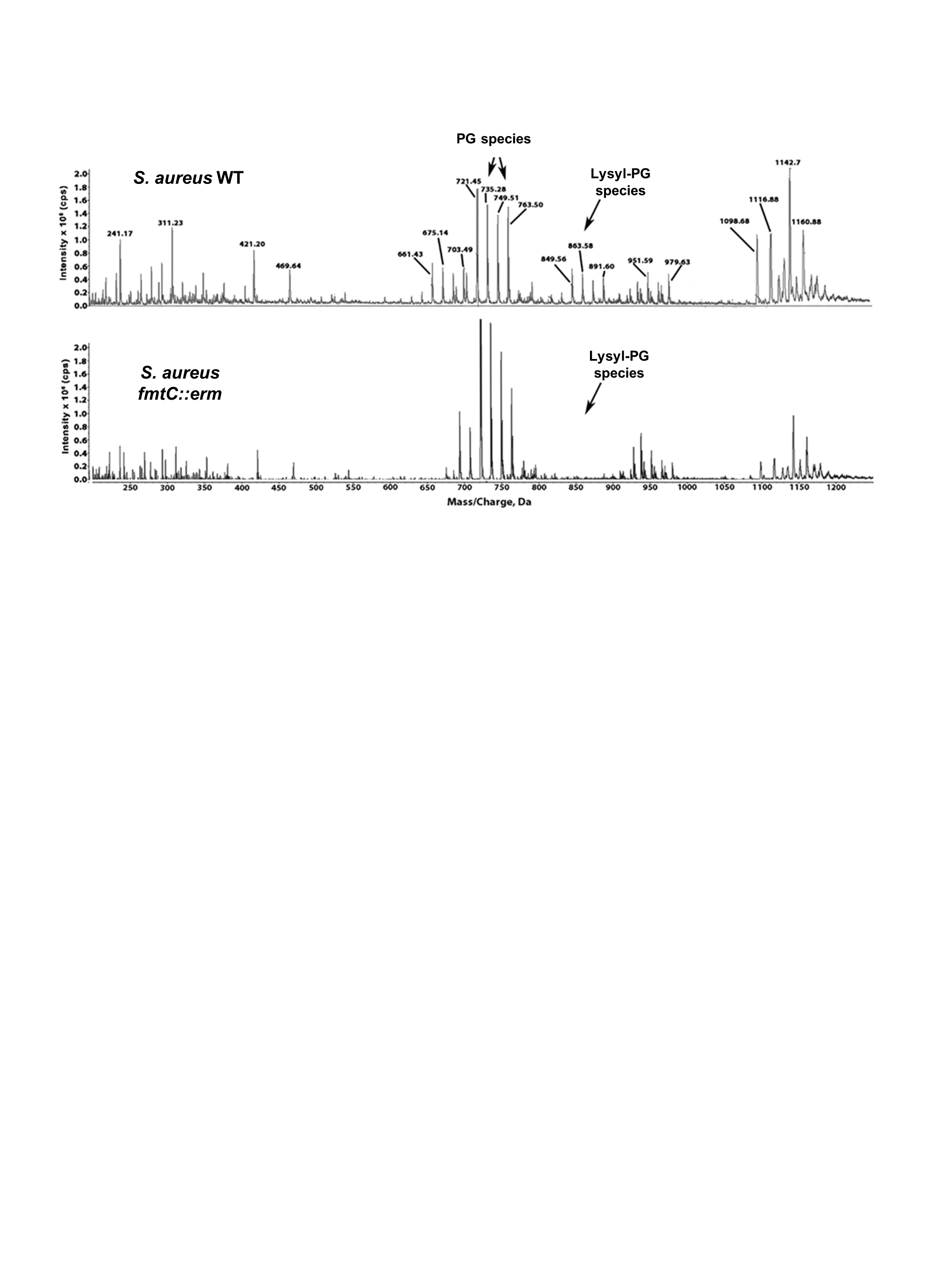

Supplement: S6 Fig — Mass spectra showing that the fmtC::erm mutant strain of SA (bottom panel) retains all other major SA lipid moieties except for lysyl-PG species. (TIF) [file ppat.1008443.s006.tif]

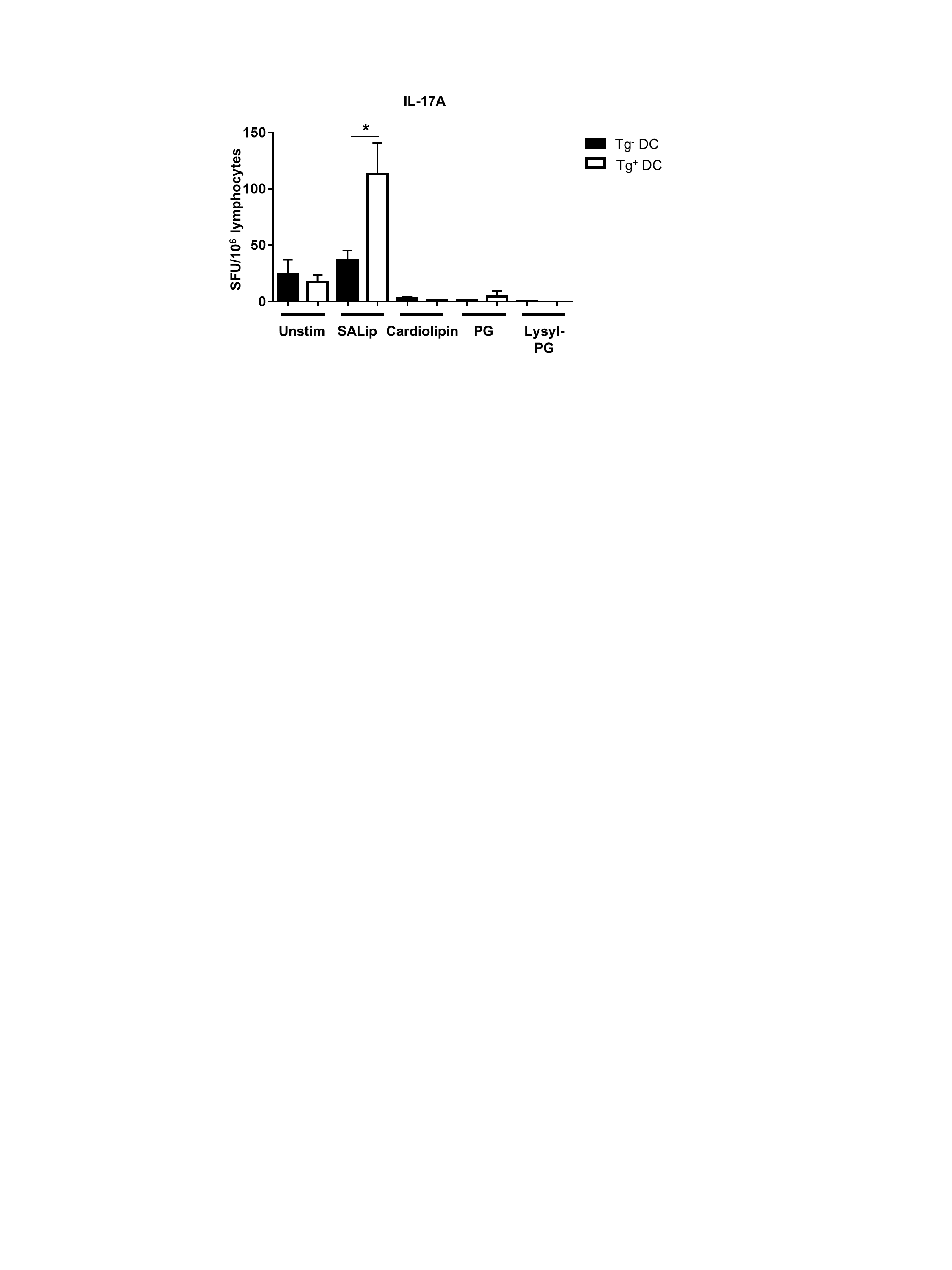

Supplement: S7 Fig — hCD1Tg mice were infected with 3x10^6^ CFU of SA via tail vein. Mice were euthanized at 10 days post-infection and lymphocytes from pooled peripheral lymph nodes were put into IL-17A ELISPOT. Data representative of 2 independent experiments with n = 4 mice per experiment. *p<0.05 using two-way ANOVA with Tukey’s posttest. (TIF) [file ppat.1008443.s007.tif]

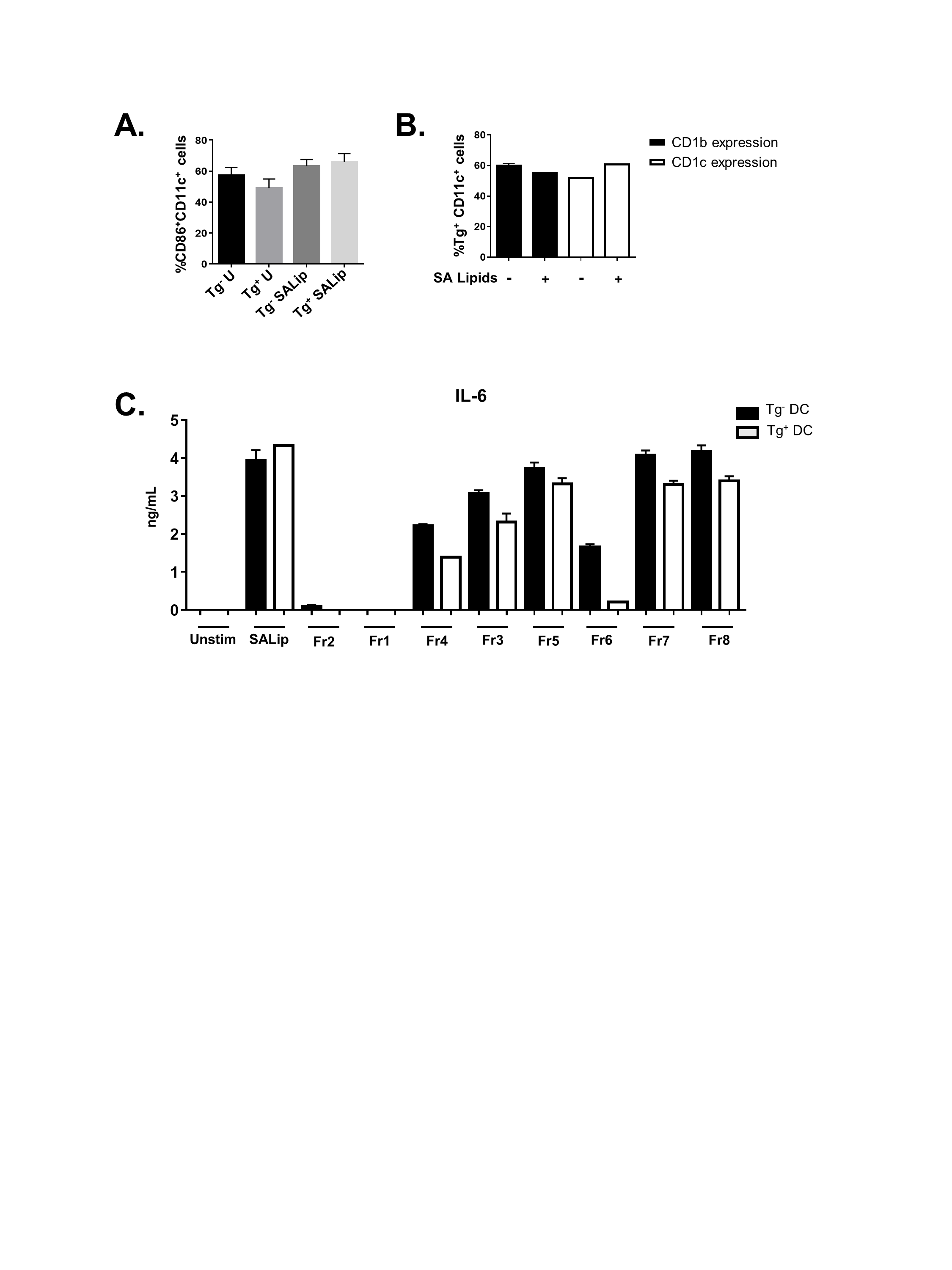

Supplement: S8 Fig — (A, B) Quantification of CD86 (A), CD1b, and CD1c (B) expression in unstimulated or SA lipid stimulated Tg- and Tg+ BMDCs. (C) Tg- and Tg+ DCs produced similar levels of IL-6 in response to SA lipids. Tg- and Tg+ BMDCs were stimulated with the indicated SA lipids/fractions for 12h and supernatants were assayed for IL-6 production by ELISA. (TIF) [file ppat.1008443.s008.tif]

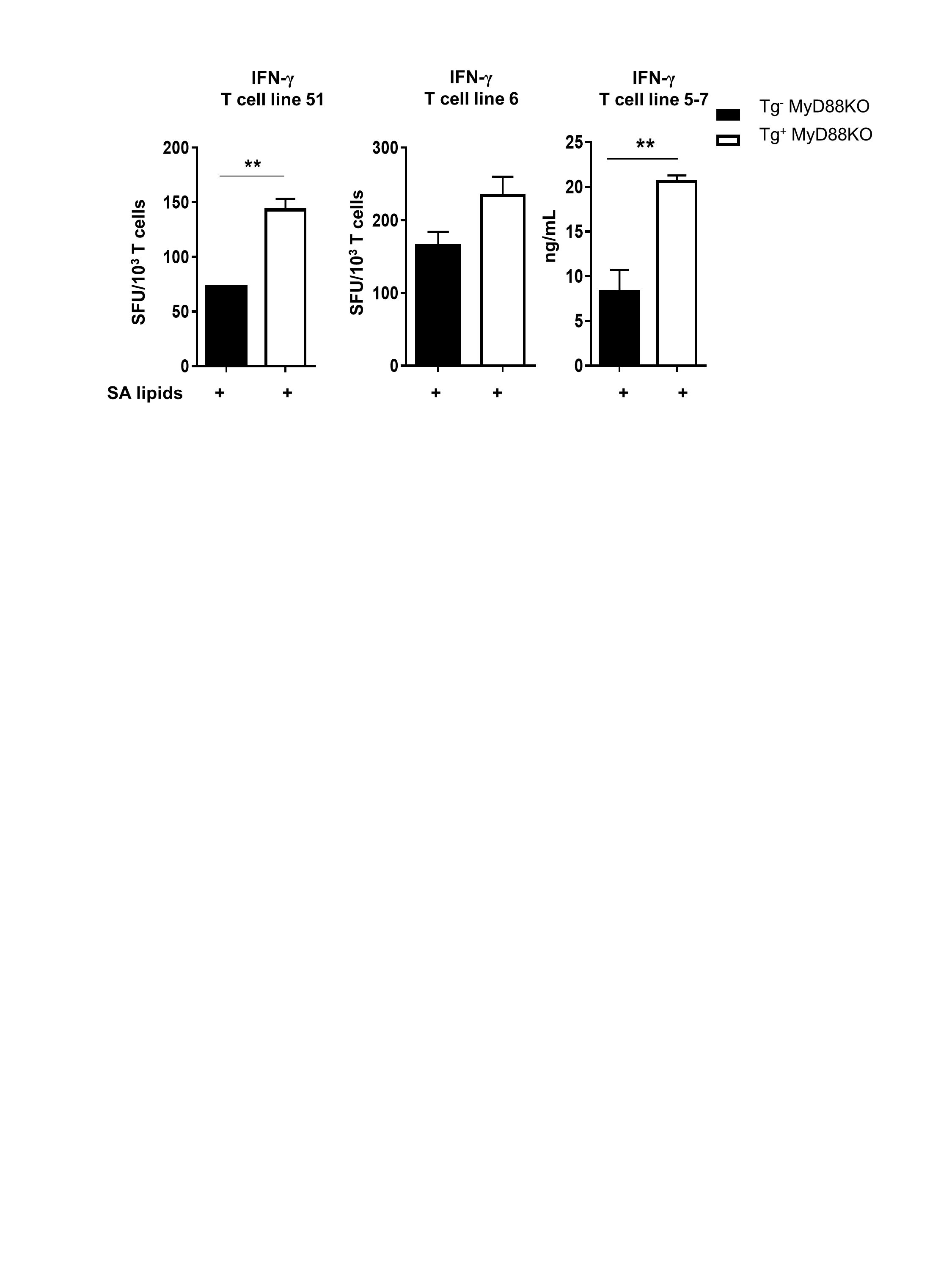

Supplement: S9 Fig — T cell lines 51, 6, and 5–7 were stimulated with Tg- and Tg+ BMDCs coated with SA lipids and lacking the MyD88 adaptor protein for NFκB signaling. Cells were co-cultured overnight (T cell line 6, 51) or for 48h (T cell line 5–7) and IFN-γ ELISPOT (T cell line 6, 51) or ELISA (T cell line 5–7) was performed. Data shown is after subtracting background from Tg- or Tg+ BMDC unstimulated conditions. Data representative of 2–3 independent experiments with each condition plated in duplicate. **p<0.01 using two-way ANOVA with Tukey’s posttest. (TIF) [file ppat.1008443.s009.tif]

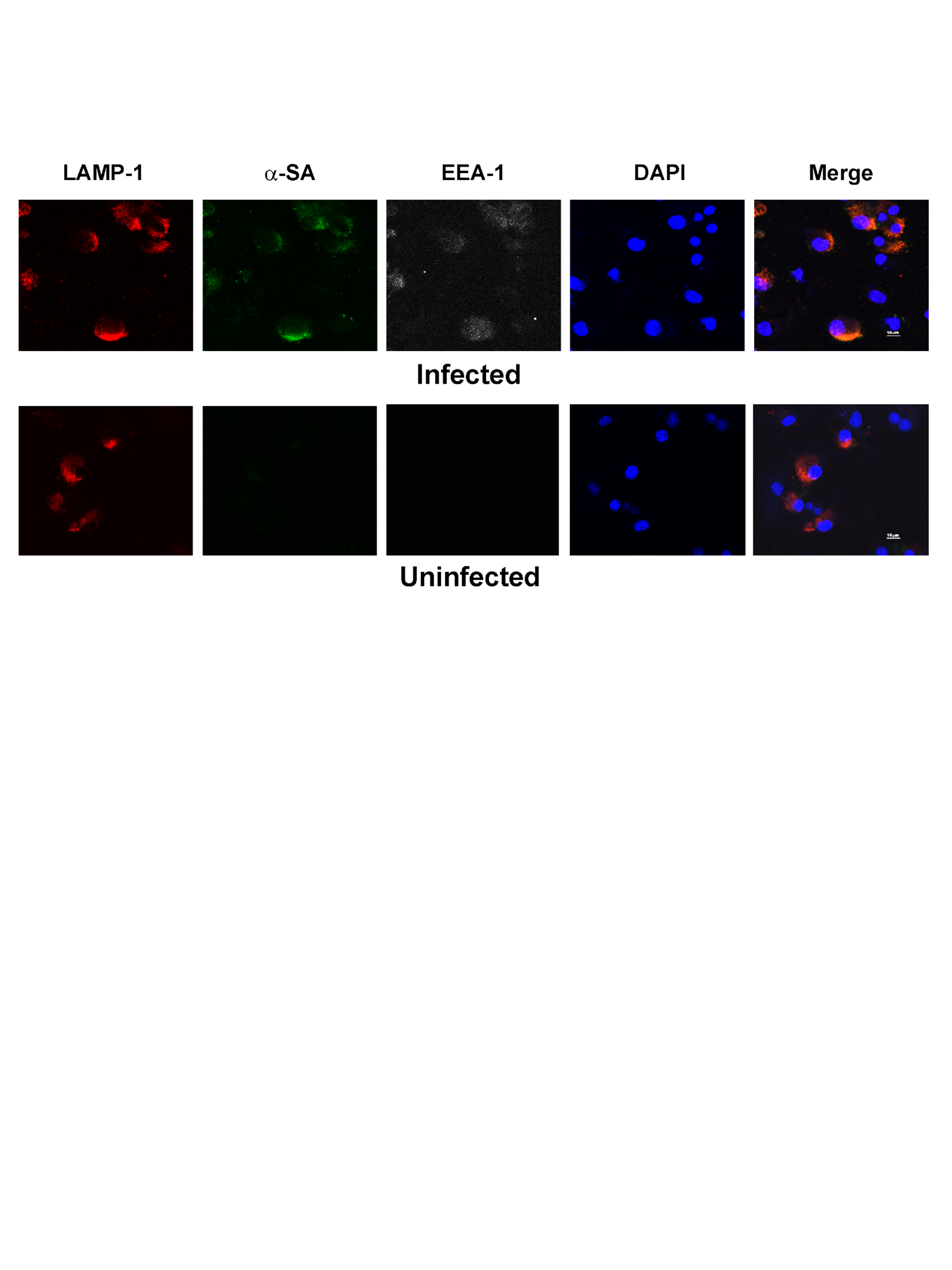

Supplement: S10 Fig — Immunofluorescence staining against LAMP1, EEA1, and SA was conducted on uninfected or SA-infected BMDCs. Data representative of 2 independent experiments with all conditions stained in triplicate. (TIF) [file ppat.1008443.s010.tif]

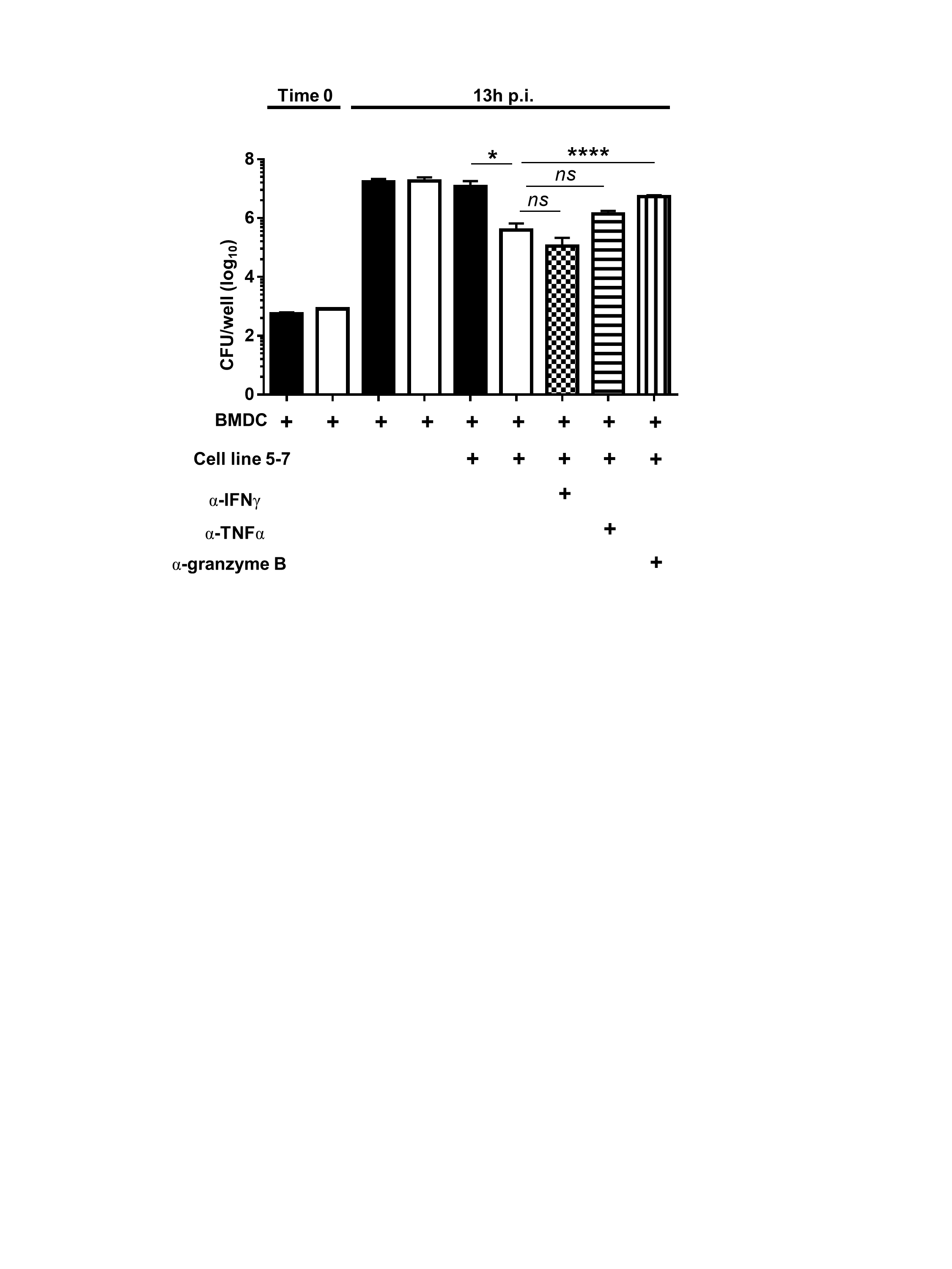

Supplement: S11 Fig — BMDCs were infected with SA as in Fig 7 and infection proceeded for 13h. Some conditions included antibody against IFN-γ or TNF-α or granzyme B in each well. Cells were lysed in the wells and bacteria pelleted and plated for CFU enumeration. All conditions were plated in triplicate. Data representative of 3 independent experiments. *p<0.05 using Student’s t test. (TIF) [file ppat.1008443.s011.tif]

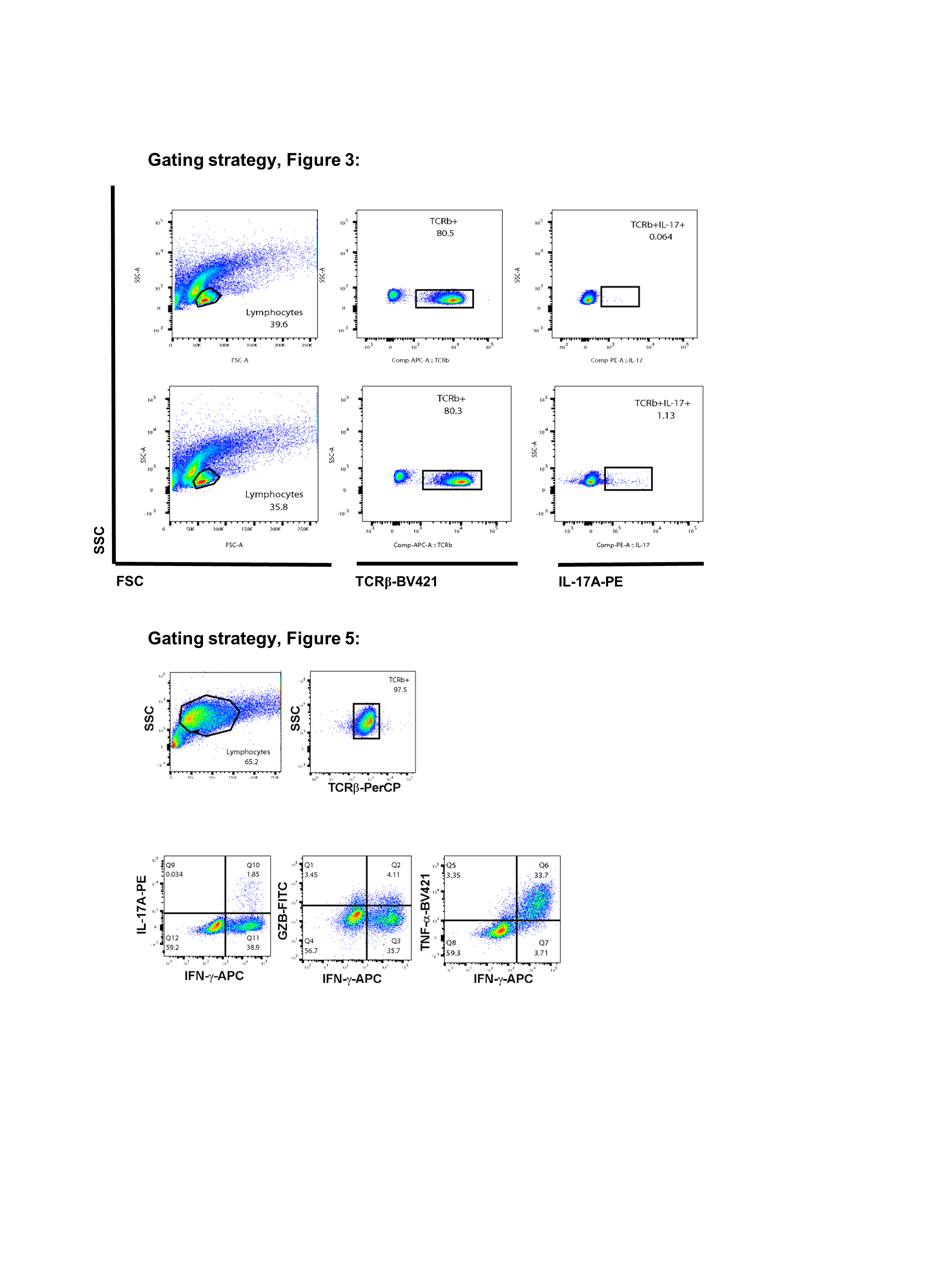

Supplement: S12 Fig — Fig 3B is gated on lymphocytes from pooled peripheral lymph nodes of hCD1Tg+ mice after depletion of endogenous antigen presenting cells and γδ T cells. Fig 5C is gated on lymphocytes from the primary T cell line CTL 5–7. (TIF) [file ppat.1008443.s012.tif]
